# Permafrost thawing as a possible source of abrupt carbon release at the onset of the Bølling/Allerød

**DOI:** 10.1038/ncomms6520

**Published:** 2014-11-20

**Authors:** Peter Köhler, Gregor Knorr, Edouard Bard

**Affiliations:** 1 Alfred-Wegener-Institut Helmholtz-Zentrum für Polar-und Meeresforschung (AWI), P.O. Box 12 01 61, D-27515 Bremerhaven, Germany; 2 School of Earth and Ocean Sciences, Cardiff University, Cardiff CF10 3AT, UK; 3 CEREGE, Aix Marseille University, CNRS, IRD, College de France, B.P. 80 Technopole de l’Arbois, 13545 Aix-en-Provence, France

## Abstract

One of the most abrupt and yet unexplained past rises in atmospheric CO_2_ (>10 p.p.m.v. in two centuries) occurred in quasi-synchrony with abrupt northern hemispheric warming into the Bølling/Allerød, ~14,600 years ago. Here we use a U/Th-dated record of atmospheric Δ^14^C from Tahiti corals to provide an independent and precise age control for this CO_2_ rise. We also use model simulations to show that the release of old (nearly ^14^C-free) carbon can explain these changes in CO_2_ and Δ^14^C. The Δ^14^C record provides an independent constraint on the amount of carbon released (~125 Pg C). We suggest, in line with observations of atmospheric CH_4_ and terrigenous biomarkers, that thawing permafrost in high northern latitudes could have been the source of carbon, possibly with contribution from flooding of the Siberian continental shelf during meltwater pulse 1A. Our findings highlight the potential of the permafrost carbon reservoir to modulate abrupt climate changes via greenhouse-gas feedbacks.

## Abstract (web summary)

Ice core records show evidence for an abrupt, and thus far unexplained, increase in atmospheric CO_2_ levels ~14,600 years ago. Here, the authors combine ice core data, a precisely dated decline in atmospheric ^14^C and numerical simulations, and propose thawing permafrost as a possible source of this event.

## Graphical abstract

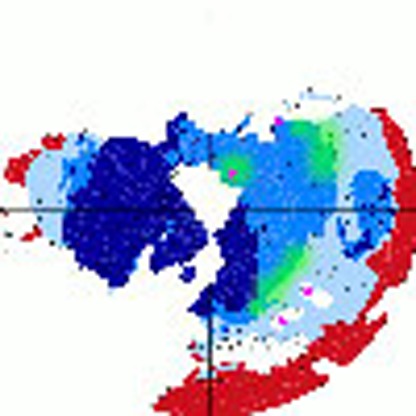

Changes in the global carbon cycle during the last deglaciation are so far not completely understood. However, based on the data and model-based interpretation, the emerging picture indicates that the rise in atmospheric CO_2_ of ~45 p.p.m.v. during the first half of the deglaciation (~1 p.p.m.v. per century) was probably fuelled by the release of old, ^13^C- and ^14^C-depleted deep ocean carbon^1^,^2^. The processes responsible for CO_2_ rise have changed dramatically with the beginning of the Bølling/Allerød (B/A) ~14,600 years before present (~14.6 kyr BP). Here the abrupt CO_2_ rise recorded in the EPICA Dome C (EDC) ice core^3^,^4^ was six times faster than before, about 10 p.p.m.v. in 180 years or ~6 p.p.m.v. per century (Fig. 1). Atmospheric CH_4_ rose by 150 p.p.b.v. between 18.5 and 14.6 kyr BP and then by the same amount again, but within centuries, around the onset of the B/A. The changes in both greenhouse gases (GHG) imply that a ratio of both changes ΔCH_4_/ΔCO_2_ is a factor of five larger around 14.6 kyr BP than during the previous four millennia. Such a change in the ratio ΔCH_4_/ΔCO_2_ might be the first indication that the wetlands identified^5^ as the main contributor to the rapid rise in CH_4_ at the onset of the B/A might also have contributed to the abrupt rise in CO_2_ at that time.

Although this analysis of CH_4_ and CO_2_ changes gives some first ideas on the potential cause of the abrupt CO_2_ rise around the onset of the B/A, its ultimate source was so far not identified. The *δ*^13^C signature of terrestrial or marine carbon sources are different and might allow some source detection. However, the data uncertainty and density of the atmospheric *δ*^13^CO_2_ record did so far not allow such an identification^6^. A high-resolution U/Th-dated time series of atmospheric Δ^14^C derived from Tahiti corals^7^ over that event offers now some new and independent insights on the exact timing and magnitude of the carbon release event and brings some suggestions on its potential origin.

Here we show that the synchronous change in atmospheric Δ^14^C and CO_2_ derived from the Tahiti and EDC data sets at the onset of the B/A can be explained by the same process and suggest permafrost thawing being this process. We finally examine the climate impact of the GHG changes around 14.6 kyr BP using a state-of-the-art-coupled Earth system model. Special focus of these investigations is the imprint of the GHG changes on the Antarctic temperature signature and the relevance of these changes for the interpretation of bipolar climate linkages during abrupt climate changes^8^.

## Results

### Atmospheric Δ^14^C and ice core CO_2_

The new coral-based atmospheric Δ^14^C record from Tahiti^7^ shows a prominent decline around 14.6 kyr BP, an anomaly not visible in the IntCal13 Δ^14^C stack^9^ (Fig. 2). For comparison, we briefly discuss specific details related to IntCal13 and what other ^14^C archives record at that point in time: After 13.9 kyr BP IntCal13 is based on tree rings with very little variability. For older samples, however, the various archives differ by more than the measurement errors. A Δ^14^C anomaly similar to the Tahiti data can be seen in speleothems from Bahamas^10^ (Fig. 2). The anomaly is not seen in speleothems from the Hulu Cave^11^ or in the marine sediments from Cariaco^12^ (Fig. 2). The Cariaco record bears some problems—therefore, a part of it has been excluded by the IntCal13 group specifically during the Heinrich 1 event, that is, just before the B/A^9^. Necessary corrections of speleothem ^14^C data for its dead carbon fraction (DCF) introduce large uncertainties to atmospheric Δ^14^C based on them^9^. Furthermore, the DCF is not constant but depends itself on climate^13^ making the speleothems an archive difficult to interpret, especially during rapid climate changes. The signal might thus potentially be smoothed out in Hulu, as the DCF acts as a low-pass filter. The best recorder of atmospheric Δ^14^C available up to now might be the terrestrial plant material derived from Lake Suigetsu^14^. Here no corrections for the reservoir effect or for DCF are necessary. The Lake Suigetsu data, however, are rather scattered over the time interval of interest, show a steeper decline in Δ^14^C than IntCal13, but neither strongly support IntCal13 nor Tahiti (Fig. 2). Altogether, the evidences from Δ^14^C data are mixed and further data are necessary for a conclusive interpretation.

The coral-based Δ^14^C record from Tahiti is corrected for a reservoir age^15^ of constantly 300 ^14^C years^7^ to be interpreted as atmospheric Δ^14^C. In principle, the reservoir age might change over time, mainly due to ocean circulation changes. However, simulations with three different models^16^,^17^,^18^ suggest that the reservoir age is relatively stable in the central Pacific around Tahiti for various ocean circulation changes (see Supplementary Note 1 for details). We therefore assume that reservoir ages did not change over the last 15 kyr in the central low-latitude Pacific and the Δ^14^C signal based on Tahiti corals is not based on local effects but indeed a recorder of atmospheric Δ^14^C changes.

We date the start of this Δ^14^C decline seen in the Tahiti data with two different approaches (Fig. 2, methods) with a 1*σ* uncertainty of less than a century to 14.6 kyr BP and calculate a Δ^14^C decline of ~55‰ within 200 to 250 years. Having already excluded changes in reservoir age, we are left with either a modified carbon cycle or reduced ^14^C production rates as potential process explaining the Tahiti Δ^14^C data. On the basis of available ^10^Be, data changes in ^14^C production rates cannot convincingly explain the Δ^14^C data (Supplementary Fig. 1, Supplementary Note 1). All our tests therefore indicate that the Tahiti Δ^14^C drop at 14.6 kyr BP is caused by carbon cycle changes. This is our working hypothesis on which all else is based on, but note that its failure cannot entirely be ruled out.

Carbon cycle changes responsible for the Δ^14^C anomaly would also leave their imprints on atmospheric CO_2_. We can therefore use the absolute U/Th-dated Δ^14^C from the Tahiti corals as an independent time constraint on the atmospheric CO_2_ rise. This is a novel new approach to synchronize atmospheric Δ^14^C and atmospheric CO_2_, because ice cores archive only a smoothed version of the atmospheric concentrations making an exact dating of the abrupt change in atmospheric CO_2_ very difficult^6^. Furthermore, firnification and gas enclosure are still not completely understood^19^, and the age difference between ice matrix and embedded gases complicates gas chronologies^3^. On the most recent chronology^4^, AICC2012, CO_2_ measured *in situ* in the EDC ice core rises by 10 p.p.m.v. between 14.81 and 14.68 kyr BP. This is more than a century faster when compared with previous chronologies^3^ (Figs 3a and 4b), but might in detail be revised even further once the most recent understanding of firnification is applied^20^. Atmospheric changes in CO_2_ need to have happened even more abruptly than what is recorded in ice cores^6^.

Here we use the Tahiti Δ^14^C as an independent age constraint for the start of the carbon cycle changes (14.6 kyr BP). Recently, others^21^ have shown that the rise in atmospheric CH_4_ and in temperature in Greenland are near synchronous (5±25 years) at the onset of the B/A warming. From previous GHG records measured at the EDC ice core^22^, it is known that CO_2_ and CH_4_ also rise synchronously at 14.6 kyr BP. Combining this information, we have to conclude that the rise in atmospheric CO_2_ and CH_4_ together with the rapid warming of the northern hemisphere (NH) happened at the same time, and started at 14.6 kyr BP. This is only 35 years later than the suggested age of the onset of the B/A (14.635±0.186 kyr BP, ±1*σ*) in the annual-layer counted NGRIP ice core^23^,^24^, well within the dating uncertainty of GICC05 (Fig. 5).

### Carbon cycle simulations

A release of 125 Pg of C into the atmosphere within a time window of 50 to 200 years was proposed before^6^ to explain the rise of 10 p.p.m.v. in CO_2_ measured in EDC. The true atmospheric CO_2_ then shows an overshoot whose peak amplitude mainly depends on the length of the assumed release time (Supplementary Fig. 2). Furthermore, low-resolution CO_2_ time series from other ice cores with amplitudes of 17, 15 and 19 p.p.m.v. in Byrd, Taylor and Siple Dome, respectively^25^,^26^,^27^,^28^ (Fig. 3a), indicate that the true atmospheric signal had a larger amplitude than what was measured *in situ* in EDC^6^.

In our previous analysis, we also investigated atmospheric *δ*^13^CO_2_, from which the potential source of the released carbon might have been identified. The new compilation of ice core *δ*^13^CO_2_ data published in the mean time^1^ offers a new look on the information contained in that record. This *δ*^13^CO_2_ compilation is now based on data from EDC, EPICA DML and Talos Dome. In our time window of interest (15–14 kyr BP), however, only data from EDC were obtained with some new data points adding to the previous record. This revised *δ*^13^CO_2_ record shows a drop by 0.1‰ around 14.7 kyr BP followed by a subsequent rise by ~0.2‰ at 14.4 kyr BP (Fig. 3b). Distinguishing terrestrial from marine carbon sources for our carbon release leads in our best-guess scenarios (see below how that was chosen) to either a drop in *δ*^13^CO_2_ of 0.4‰ or less than 0.1‰ in the true atmospheric signal, respectively, but only to −0.15‰ and less than −0.05‰ in a time series that would be recorded in EDC (Fig. 3b). Both marine- and terrestrial-based *δ*^13^CO_2_ simulations fall within the uncertainties of the measurements before 14.6 kyr BP. The small rise in *δ*^13^CO_2_ after 14.4 kyr BP, however, indicates that directly after the onset of the B/A other processes released less *δ*^13^C-depleted carbon to the atmosphere, for example, CO_2_ outgassing from warm oceanic surface waters. On the basis of the data uncertainty of *δ*^13^CO_2_ in EDC, it is still impossible to clearly identify if the released carbon was of marine or terrestrial origin.

Using the same carbon cycle model^6^, we repeat simulations of carbon release with special focus on ^14^C. The model dynamic with respect to ^14^C was extensively evaluated (Supplementary Note 2). We assume a depletion in ^14^C of the released carbon with respect to the atmosphere (Δ(Δ^14^C)) between −50 and −1,250‰. This range in the ^14^C anomalies covers carbon sources from the mean terrestrial biosphere potentially released by shelf flooding^6^ (−50‰), suggested signatures of old carbon of Pacific intermediate waters as measured off Baja California^29^ (−400‰) and Galapagos^30^ (−700‰) to a maximum effect of ^14^C-free carbon (−1,250‰). The Shelf Flooding Hypothesis is explained in detail in the next section, and some more details on our assumptions on ^14^C are found in the methods.

The highest-simulated anomalies in atmospheric Δ^14^C are obtained for short release times reaching −100‰ for 50 years and the largest Δ(Δ^14^C) of −1,250‰ (Fig. 4c). The amplitudes drastically decline with longer release time towards less than −35‰. Δ^14^C anomalies are significantly smaller if Δ(Δ^14^C) was −700‰ or less (Fig. 4c). Because of the distinct dynamics of the Δ^14^C data, release times shorter than ~110 years are at odds with the Tahiti ^14^C reconstruction. Simulated anomalies in atmospheric carbon records are nearly identical for Atlantic meridional overturning circulation (AMOC) in the strong or weak mode (Methods, Fig. 4c, Supplementary Fig. 3). Combining the information from both the ice core data and our analysis of the Tahiti Δ^14^C data leads to a range of scenarios with carbon release times between ~110 and 200 years in which model results and data agree (Fig. 4c). The range of possible scenarios fulfilling the data constraints also includes some with Δ(Δ^14^C) between −700 and −1,250‰, and so we cannot entirely exclude the possibility that the released carbon still contains some ^14^C. From these possible scenarios, we selected the one with the longest release time of 200 years to be our best-guess scenario, because short release times lead to higher amplitudes in atmospheric CO_2_, which are not supported by other ice core data. This scenario pinpoints to a Δ(Δ^14^C) of −1,250‰ resulting in peak amplitudes of −42‰ in atmospheric Δ^14^C (Fig. 4c) and of +22 p.p.m.v. in atmospheric CO_2_ (Fig. 4d). The depletion in ^14^C necessary for the model output to agree with the data implies that the shelf flooding hypothesis connected with meltwater pulse 1A (MWP-1A)^6^,^31^ seems at a first glance to be in disagreement with the Tahiti-based atmospheric Δ^14^C reconstructions (Fig. 4c). We discuss details on a potential contribution connected with MWP-1A further below. The release of deep ocean carbon, although so far not suggested to play a role during this rapid CO_2_ rise, might only potentially be responsible here, if water masses are detected, which are even more depleted in ^14^C than what is known until now^29^,^30^.

**Figure 1:**
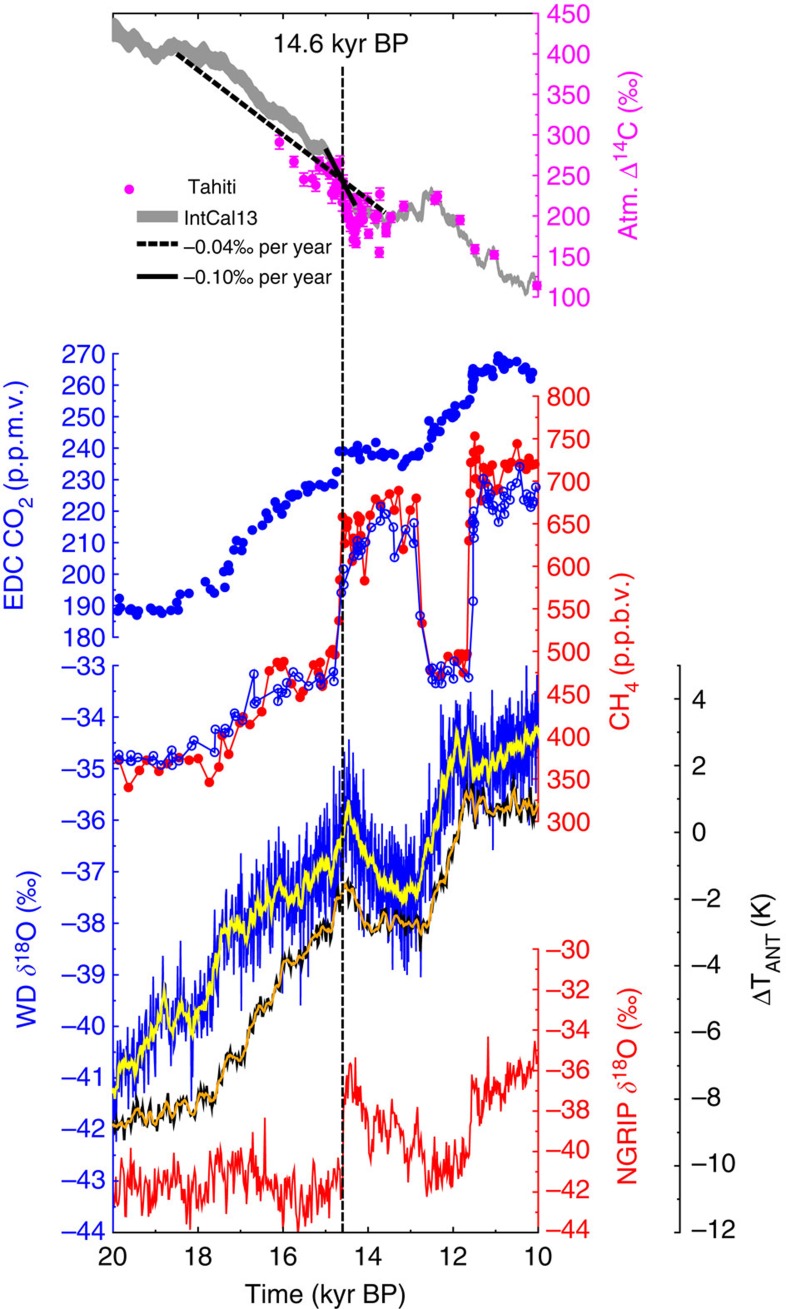
Relevant ice core and Δ^14^C data during Termination I. Atmospheric Δ^14^C based on Tahiti corals (magenta circles, mean ±1*σ*)^7^ or IntCal13 (grey area, ±1*σ* uncertainty band around the mean)^9^, the latter including linear trends with −0.04‰ per year or −0.10‰ per year; CO_2_ from EDC (blue filled circles)^1^,^22^,^68^; CH_4_ from EDC (blue)^22^ and Greenland (red)^69^; WAIS Divide (WD) *δ*^18^O (original (blue) and 100 years running mean (yellow))^54^; stack of calculated temperature change^3^ ΔT_ANT_ from the five East Antarctic ice cores EDC, EPICA DML, Vostok, Dome Fuji and Talos Dome (original (black) and 100 years running mean (orange)); NGRIP^70^ *δ*^18^O. All Greenland records on GICC05 (ref. 23), all EDC records on AICC2012 chronology^4^, WD and ΔT_ANT_ on their own independent chronology.

The Tahiti Δ^14^C data show an excursion from the long-term declining trend of IntCal13 (ref. 9) (Figs 1 and 4a). Depending on the time window of interest, IntCal13 might be approximated by a linear fit with a slope of −0.04‰ per year (the whole Mystery Interval, 19–14 kyr BP) or −0.10‰ per year (15.0–14.3 kyr BP), respectively. These long-term changes are probably caused by a mixture of changes in ^14^C production rate and the carbon cycle^32^. While we are able to force our model with changing ^14^C production rates (Supplementary Fig. 4), all relevant processes in the carbon cycle are not yet identified. We therefore compare the Δ^14^C data with our original simulation results based on constant ^14^C production rate, but also with some results that are corrected for the trend seen in IntCal13. If corrected accordingly, our best-guess scenario finally meets the amplitude in the Tahiti Δ^14^C data (Fig. 4a,c).

### Evidence for permafrost thawing

The synchronicity of the NH warming and the carbon cycle change together with our suggested hypothesis for the injection of nearly ^14^C-free carbon into the atmosphere make permafrost thawing and a subsequent release of old soil carbon a prominent candidate to explain the atmospheric carbon records. The age of carbon stored in permafrost soils during glacial times is unknown. Throughout the last glacial cycle Greenland and the whole NH was perturbed by the rapid warming of Dansgaard/Oeschger (D/O) events^33^. However, during the last 80 kyr, only D/O event 12 around 47 kyr BP reached in a temperature reconstruction for the site of the NGRIP ice core in Greenland similar high temperatures as the B/A (Fig. 6b). In this NGRIP, temperature time series D/O event 2 around 23 kyr BP was rather weak and short, but D/O event 3 at 28 kyr BP reached with −36 °C nearly the temperature of −33 °C of the B/A^33^ (Fig. 6b). We assume that most of the NH follow this temporal changes in temperature observed for Greenland, although with warmer temperatures closer to the freezing point further south. It might then be that large areas of the NH were permanently frozen after D/O event 3, thus about 13 kyr before thawing induced by the onset of the B/A. The Δ^14^C of that permafrost carbon would be depleted by −900‰ with respect to atmospheric Δ^14^C during release around 14.6 kyr BP (Fig. 6a). However, soil carbon might age significantly in high latitudes before freezing, for example, present day North American peatlands are up to 17-kyr old^34^. Such soil ageing reduces ^14^C even further. If the precursor material of the permafrost soil carbon was photosynthetically produced during D/O event 12 around 47 kyr BP (the next preceding period comparable in temperature to the B/A, Fig. 6b), it would be essentially free of ^14^C and depleted with respect to atmospheric Δ^14^C by nearly −1,250‰ (Fig. 6a). Permafrost thawing would then contain a depletion in Δ^14^C, which is more negative than for all other suggested processes^6^,^29^,^30^. An alternative scenario based on the destabilization of gas hydrates, which also contain ^14^C-free carbon, can be rejected based on CH_4_ isotopes^35^,^36^,^37^.

For the present day, a rise in global mean temperature by 5 K, which because of polar amplification might represent a northern high latitude warming of 10 K, was proposed to lead to the release of more than 130 Pg of soil carbon from permafrost thawing within 200 years^38^. Greenland ice core data^33^ and simulations^39^ suggest that temperatures in the B/A rose by 10–15 K to near preindustrial levels in central Greenland and throughout most of the NH land areas. A large inert terrestrial carbon pool consisting of permafrost soils containing 700 Pg more C at the Last Glacial Maximum (LGM) than at present day has been proposed^40^, which needs to release its excess carbon during deglaciation. The areal extent of continuous permafrost at LGM (Fig. 7) was calculated from models^41^ in PMIP3 to 26 × 10^12^ m^−2^, agrees with reconstructions^42^, and is twice as large as for preindustrial times^41^.

Previously, methane isotopes^36^ suggested that a rise in boreal wetland CH_4_ emissions by +32 Tg CH_4_ per year would explain the CH_4_ rise into the B/A. These findings^36^ have been challenged by new methane isotope data^37^, but so far no revised CH_4_ emissions from boreal wetlands have been calculated for the B/A. An alternative interpretation^5^ of the CH_4_ cycle based on its interhemispheric gradient suggests that the rise in CH_4_ by 150 p.p.b.v. at the onset of the B/A was largely driven by the increase in CH_4_ emissions from both tropical (+35 Tg CH_4_ per year) and boreal (+15 Tg CH_4_ per year) wetlands. The CH_4_ change at the onset of the B/A is thus clearly dominated by tropical wetlands and its conclusive interpretation is beyond the scope of this study. However, the rise in CH_4_ emissions from boreal wetlands is nearly identical to the rise in emissions of up to +14 Tg CH_4_ per year projected from deep permafrost thawing of the next century^43^. If this rise in the boreal CH_4_ flux is integrated over the 200-year time window of our carbon release scenario, a total of 3.0 Pg of CH_4_ (or 2.25 Pg C in the form of CH_4_) might have been released. This is ~2% of our total estimated carbon emissions of 125 Pg C, and in line with an expert assessment on the future vulnerability of permafrost^44^ estimating that 2–3% of carbon released by thawing might enter to the atmosphere in the form of CH_4_. Although the contribution from boreal wetlands to the CH_4_ rises at the onset of the B/A is small, the nearly ^14^C-free signature connected with our proposed permafrost thawing might be tested by ^14^C measurements on CH_4_ derived from ice cores^45^.

So far, we suggested that NH permafrost is the responsible source of the released carbon. In the following, we hypothesize which region might have been affected in detail by permafrost thawing and how this can be tested in future studies. The PMIP3-based map on the LGM permafrost extent clearly indicates that the largest areas with continuous permafrost are found in northern Siberia (Fig. 7). Thus, evidences of permafrost thawing connected to the NH warming should be expected in outflow originated from the southern edge of the LGM permafrost area (around 40–50°N), which thawed first. A lot of these areas are drained via the Amur river into the Sea of Okhotsk and into coastal seas towards the south (Caspian and Black Sea). Indeed, a combination of terrestrial biomarkers that clearly indicate the thawing of permafrost was found at the onset of the B/A in a sediment core drilled in the Black Sea that records the drainage from the Fennoscandian Ice Sheet^46^. Here variations in the normalized concentrations of different long-chain molecules provide information on changes in the abundance of peat-forming plants. Such data are useful indicators for the variation of permafrost thawing and of wetland extension as well as for fluvial periglacial soil erosion in its drainage basin. In details, this study^46^ provides evidence that the permafrost melting was very intense only during the initial part of the Bølling corresponding indeed to the sharp NH warming.

The map (Fig. 7) also shows that a large fraction of the Siberian shelf in the Arctic Ocean was during the LGM covered by permafrost. It might thus be possible that MWP-1A, which was recorded as a rise in sea level at Tahiti^31^ from about −105 to −85 m between 14.65 and 14.31 kyr BP might be partially responsible for the carbon release as initially suggested^6^. The flooding of continental shelves was also proposed to contribute to the CH_4_ rise during deglaciation and during D/O events^47^. In our earlier study^6^, we proposed that mainly the flooding of the Sunda Shelf followed by tropical rain forest decay might have been responsible for the carbon release. This shelf flooding scenario was here addressed with a Δ(Δ^14^C) of −50‰ for mean terrestrial carbon, which failed to meet the Δ^14^C data. In our earlier study^6^, we also discussed that the existing time series of sea level change suggest that before MWP-1A the shelves were last flooded around 30 kyr BP, 15 kyr earlier, leaving ample time for ^14^C in permafrost carbon on the shelf to decay and to produce a Δ(Δ^14^C) in the released carbon of down to −900‰ (Fig. 6). Most recent sediment data^48^ on iceberg discharge in Antarctica during Termination I found a significant Antarctic contribution to MWP-1A. Fingerprint analysis^49^ of different water sources for MWP-1A indicate that sea level would rise locally by up to 50% above global average on the Siberian Shelf for freshwater released in Antarctica. When considering the source-depending overprint^49^, we calculate, based on the present day bathymetry^50^, a maximum areal extent of 0.4 × 10^12^ m^−2^ of the Siberian Shelf, which might have been flooded by MWP-1A. This is the same order of magnitude as the present day Siberian Yedoma deposit extent^51^ from which an organic carbon content of 30–140 Pg C has been proposed^51^. Coastal erosion and sub-sea permafrost release in Arctic Siberia are also observed for modern times^52^ with a Δ^14^C signature of the released organic carbon as low as −800‰. Modern organic carbon content in Eurasian Arctic^53^ river runoff have Δ^14^C ages of up to 10 kyr. All these modern data indicate that old carbon in permafrost exists nowadays, and potentially was more abundant and older during glacial times.

In which region the thawing of permafrost finally happened might be verified by future ^14^C measurements on terrigenous organic material that are retrieved from marine sediments in the suggested coastal seas. It will then be possible to finally attribute the size of the released carbon to either a pure thermodynamically thawing at the southern edge of the permafrost area or to a contribution from flooding the Siberian Shelf during MWP-1A.

## Discussion

The rapid CO_2_ rise at the onset of the B/A is contained with different amplitude in various ice cores (Fig. 3a). However, the uncertainty in the proposed age distribution of the CO_2_ in EDC is still large^6^ (Supplementary Fig. 2c) and the assumed carbon release history and the applied carbon cycle model influence the amplitude of the proposed true atmospheric CO_2_ rise. Future CO_2_ measurement from the WD ice core^54^ might refine some of these aspects. The WD ice core has an order of magnitude higher present day accumulation rate than EDC (20 versus~3 g cm^−2^ per year), thus offers temporally higher resolved gas records. A potential WD CO_2_ record still needs to be corrected for the smoothing during firn enclosure, although this effect will be a lot smaller than for EDC. Only by considering the 20% uncertainty in the mean exchange time of CO_2_ before enclosure in EDC (Supplementary Fig. 2c) would result in a CO_2_ release and an amplitude in the true atmospheric CO_2_ rise, which are also 20% smaller than in our best-guess scenario, for example, releasing 100 instead of 125 Pg C leading to a true atmospheric CO_2_ amplitude of 18 instead of 22 p.p.m.v. (Supplementary Fig. 2d). Results are then still within the range given by the EDC CO_2_ data (Supplementary Fig. 2d). Furthermore, we calculate that a 33% reduction in the proposed carbon released to the atmosphere (85 instead of 125 Pg C) still fulfils the data constrains given by the Tahiti Δ^14^C record.

Other rapid CO_2_ rises are detected in EDC at the end of the Younger Dryas and in Marine Isotope Stage (MIS) 3 in other ice cores^55^. Whether they are also caused by permafrost thawing is not investigated here. However, these other rapid CO_2_ jumps are not always connected to a warming of the NH. Furthermore, the missing of ^14^C-depleted CH_4_ during the CO_2_ rise at the end of the Younger Dryas around 12 kyr BP^45^ suggests that other processes are responsible. Moreover, our suggested old soil carbon release during permafrost thawing at 14.6 kyr BP, requires that the same carbon sources were not tapped during other events within Termination I. Again, ^14^C measurements on terrigenous material might clarify how old the carbon released from permafrost was or if earlier CO_2_ rises might already have consumed the old, ^14^C-depleted carbon.

Our study suggests that for Termination I, abrupt warming in the NH might lead to massive permafrost thawing, activating a long-term immobile carbon reservoir. The abruptly released carbon then amplified the initial warming as a positive feedback. Our best-guess scenario generates together with the rise in the other two important GHG CH_4_ and N_2_O a radiative forcing^6^ of ~0.7 W m^−2^. It is important to quantify the feedback of this GHG forcing on climate to better understand the impact of the abrupt GHG changes during the last deglaciation. Since the abrupt GHG changes are contemporaneous with the onset of the B/A (in the North) and the beginning of the ACR (in the South), the sequence of the associated bipolar climate linkages are of particular interest.

Therefore, we have conducted transient simulations with our best-guess reconstruction of atmospheric GHG changes during the beginning of the B/A and the ACR, using the Earth System Model COSMOS^16^,^56^ in a coupled atmosphere-ocean configuration as outlined in detail in the Supplementary Note 3. To evaluate the global impacts of the GHG changes it is instructive to analyse Antarctic temperature changes, since temperature changes obtained from Antarctic ice cores have been shown to reflect global scale climate changes associated with CO_2_ variations particularly well^57^. On the basis of our climate model investigations, we find that the abrupt rise in GHG concentrations provides an important impact on the Antarctic temperature signature associated with an abrupt AMOC strengthening at the end of Heinrich Stadial 1 (Supplementary Fig. 5). This highlights a potential contribution of abrupt GHG changes on the bipolar climate signature during deglaciation. In this sense, the abrupt GHG changes would be a factor that would offset the timing of the temperature maximum leading into the ACR, compared to the onset of the B/A. As layed out in detail in the climate feedback section of the Supplementary Note 3, also smaller GHG spikes bear the potential to have a substantial effect on the Antarctic temperature response when compared with impacts caused by AMOC changes.

So far a synthetic record of Greenland temperature changes^8^ seems to indicate that rapid climate changes in the north might indeed have been a universal feature of deglaciations during the last 800 kyr. Hence, similar to the last deglaciation, abrupt permafrost thawing might have also occurred regularly during earlier terminations, although further studies are necessary here. Termination II also contains^58^ an abrupt rise in CO_2_, synchronous to a rise in CH_4_. A massive drop in atmospheric *δ*^13^CO_2_ accompanying this event^58^ is consistent with the release of *δ*^13^C-depleted CO_2_ that might indicate a terrestrial source. However, new *δ*^13^CO_2_ data^59^ did not confirm this negative *δ*^13^CO_2_ anomaly and the revised data give no indication on the source of this CO_2_ rise. A synchronous change in deuterium excess^60^, a proxy for moisture source shifts, has been used to suggest that abrupt shifts in southern westerlies might be connected with the CO_2_ rise^61^, but a compelling explanation remains elusive and further testing of permafrost thawing as a possible alternative interpretation is needed.

In conclusion, we here suggest that the processes responsible for the abrupt CO_2_ rise at the onset of the B/A is also the underlying cause for the drop seen in atmospheric Δ^14^C based on Tahiti corals. This connection offers a U/Th-dated tie point for the start of the massive release of carbon at 14.6 kyr BP. Using a carbon cycle box model, and assuming the release of 125 Pg of nearly ^14^C-free carbon, we are able to explain observed anomalies in atmospheric CO_2_ and Δ^14^C. On the basis of the ^14^C signature of the released carbon and the synchronicity to the warming of the NH, we suggest that the thawing of permafrost was this responsible process. A potential contribution from MWP-1A flooding the Siberian Shelf, which might have contained a large amount of permafrost, is also possible. Future ^14^C measurements on terrigenous material might further constrain the source region. Our interpretation not only provides conceptual insights into the source of the excursions in the atmospheric carbon records around 14.6 kyr BP, but also offers an alternative to explanations^62^,^63^ for the interhemispheric timing of the B/A and the ACR as found in ice cores from both hemispheres. Taken together, our findings highlight a potential climate feedback that might be obtained from abrupt CO_2_ release during deglaciation. This analysis furthermore indicates that the proposed carbon cycle feedback from an anthropogenic driven permafrost thawing in the near future^38^,^43^,^44^,^64^ may already have happened in a similar way in the past.

## Methods

### Analysis of the Δ^14^C data

**Figure 2:**
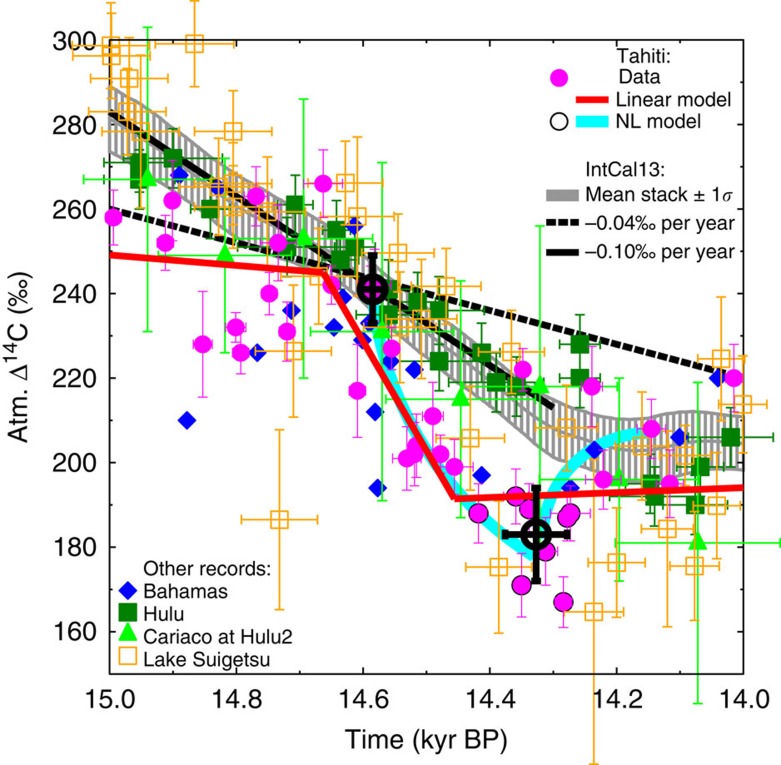
Data analysis of atmospheric Δ^14^C around 14.6 kyr BP. Atmospheric Δ^14^C based on Tahiti corals^7^ (magenta circles) and IntCal13 (grey line, ±1*σ* uncertainty band around the mean)^9^ are analysed for trends and compared with various other archives (speleothems from Bahamas^10^ (blue diamonds), Hulu Cave^11^ (dark green squares), marine sediments in Cariaco^12^ (light green triangles) plotted on revised Hulu2 age model^9^, Lake Suigetsu^14^ (orange open squares)). All individual data points are plotted with ±1*σ* in both age and Δ^14^C. IntCal13 was approximated by a linear trend with either −0.04‰ per year (black solid line) or −0.10‰ per year (black dashed line). Tahiti data were analysed for break points with two different models (see Methods). For the linear model (red lines), a statistical package was used. For the non-linear (NL) model (cyan lines), two data points at beginning of the Tahiti data Δ^14^C anomaly from the IntCal13 data and the eight points around the local minimum (black open circles) were averaged, plotted with ±1*σ* in both age and Δ^14^C (bold large black open circles) and further analysed. The anomaly in the Tahiti Δ^14^C data following the linear model is Δ(Δ^14^C)=−54±8‰ in Δ(age)=207±95 years and following the NL model: Δ(Δ^14^C)= −58±14‰ in Δ(age)=258±53 years.

For analysis of the drop in the atmospheric Δ^14^C data based on Tahiti corals, we used two different approaches (Fig. 2). First, we used a linear statistical model Breakfit^65^, which calculates the break points in time series. Breakfit searches for two linear functions that are joined at the break point. To determine the break points, the model is fitted to the data applying an ordinary least-squares method with a brute-force search for the break points. A measure of the uncertainty of the break points is based on 2,000-block bootstrap simulations, applying a moving block bootstrap algorithm with a block length of 1. We were searching for two break points in the time intervals between 16 and 13 kyr BP. The two subintervals (one for each break point) were ranging from the outer boundary next to the break point of interest to the other break point. Subintervals were finally identified after at least two iterative applications to (in kyr BP): break point 1 [15.74, 14.45] and break point 2 [14.67, 13.16]. Breakfit identified the start in the Δ^14^C drop at 14.66±0.07 kyr BP followed by its decline by 54±8‰ within 207±95 years. Because of the very distinct dynamics of atmospheric Δ^14^C including a rebound after its minimum (that is, after the carbon release to the atmosphere stopped), we also analysed the data more subjectively with a non-linear approach. Here we only calculated the mean time and mean Δ^14^C right at the start of the carbon cycle changes around 14.6 kyr BP (two data points) and at its minimum (eight data points) assuming that Δ^14^C followed a non-linear pathway between both and included a rebound thereafter. The Δ^14^C data then starts to decline at 14.59±0.04 kyr BP and stop after 258±53 years with a maximum drop of 58±14‰ followed by a rebound of atmospheric Δ^14^C. This non-linear dynamic is seen in the Tahiti data but also in our carbon cycle simulations (Figs 2 and 4a). Combining the linear and non-linear approach brings high confidence that the Δ^14^C drop started at around 14.6 kyr BP. All uncertainties are given as 1*σ*.

### Possible Δ^14^C signature of permafrost carbon

The maximum possible Δ(Δ^14^C) of carbon released from permafrost thawing is a function of age and of atmospheric Δ^14^C during time of production. From the Δ^14^C signature (IntCal13) (ref. 9) of the precursor material (atmospheric CO_2_), which varies before the B/A roughly between 250 and 550‰ (Fig. 6a), we first subtract the mean Δ^14^C value of terrestrial carbon at the LGM in the model (−50‰) before a further reduction in Δ^14^C signature is realised by the radioactive decay of ^14^C (half-life time of 5,730 years).

### Carbon cycle model

We use the carbon cycle box model BICYCLE in transient mode to simulate changes in atmospheric CO_2_ and Δ^14^C. The model setup is identical to an earlier study, which already proposed the magnitude of the CO_2_ overshoot during the B/A^6^.

**Figure 3:**
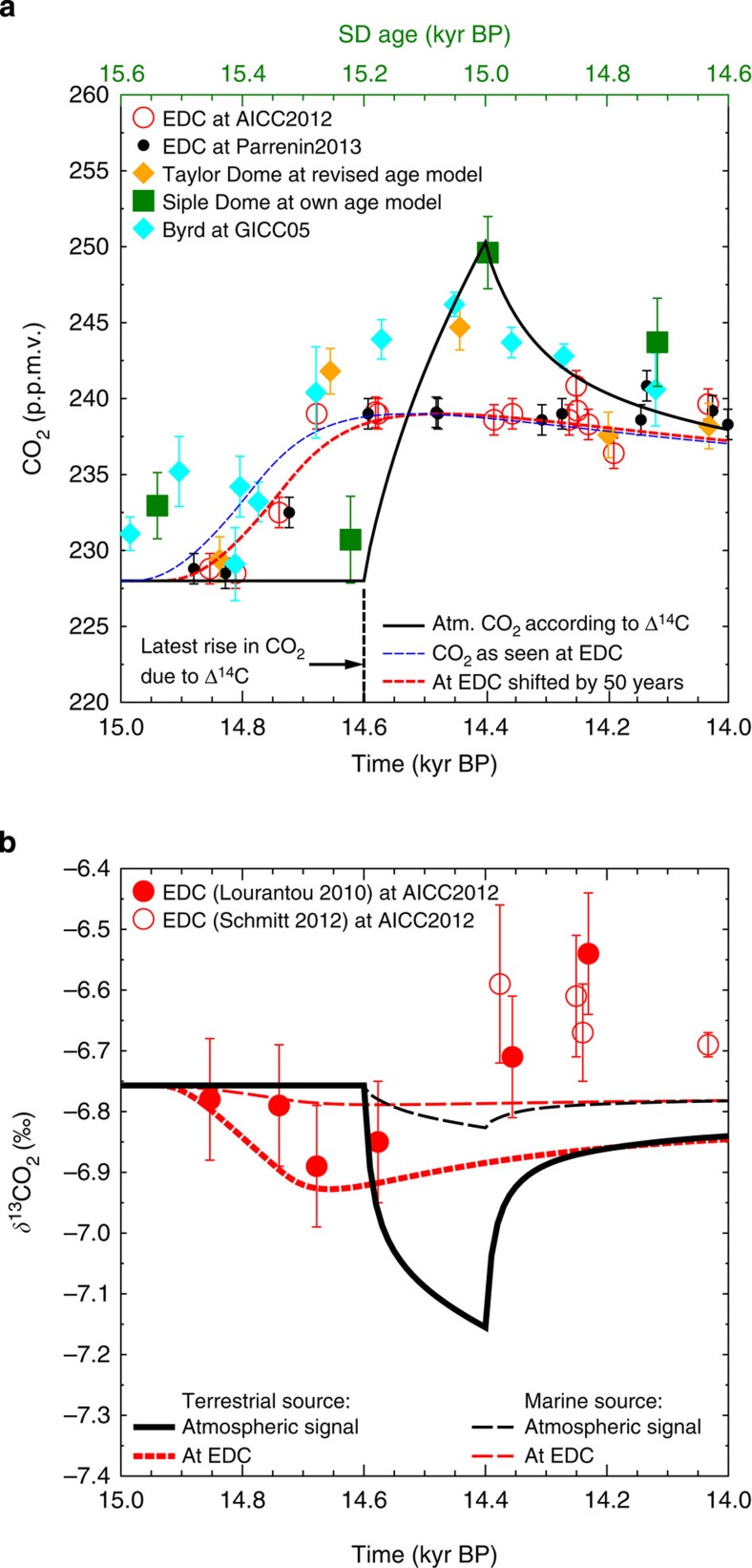
Ice core and simulated true atmospheric CO_2_ and *δ*^13^CO_2_. (**a**) Ice core CO_2_ data (±1*σ*) from EDC^1^,^22^,^68^ on two different chronologies^3^,^4^ AICC2012 and Parrenin2013, Taylor Dome on revised age model^26^,^27^, Siple Dome on own age model (top *x* axis)^27^, Byrd on age model GICC05 (refs 25, 28). Simulated true atmospheric CO_2_ in our best-guess scenario according to ^14^C data (black bold line), filtered to a signal that might be recorded in EDC (blue dashed line), shifted by 50 years to meet the EDC data (dashed red line). (**b**) Ice core *δ*^13^CO_2_ data (±1*σ*) from EDC^1^,^68^, simulated true atmospheric *δ*^13^CO_2_ of our best-guess scenario and how it would have been recorded in EDC for either terrestrial or marine origin of the released carbon, implying a *δ*^13^C signature of −22.5 or −8.5‰, respectively.

**Figure 4:**
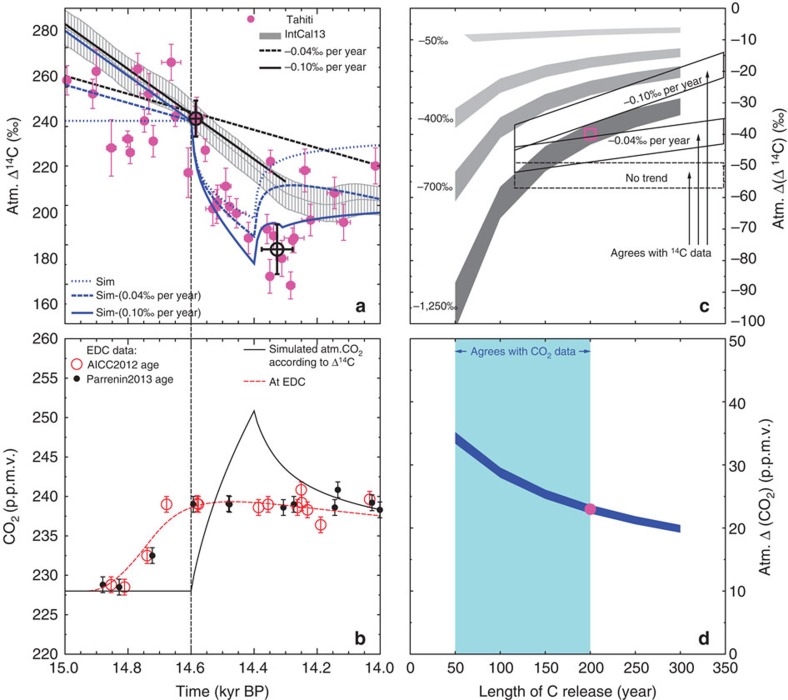
Main carbon cycle simulation results. The transient simulation results (left) showing the impact of a carbon release event on true atmospheric Δ^14^C and CO_2_ obtained with the carbon cycle model BICYCLE for the best-guess scenario are compared with the data. In sensitivity studies (right), the length of the release event and the radiocarbon signature Δ(Δ^14^C) of the released carbon are constrained by the data. (**a**) Atmospheric Δ^14^C data from Tahiti corals^7^ (magenta, mean ±1*σ* in both age and Δ^14^C) and IntCal13 (ref. 9) (grey band, mean ±1*σ*) data. Black bold circles denote start and stop (±1*σ*) of carbon release in the non-linear model of the Tahiti data interpretation. The vertical black dashed line marks the estimated started of carbon release at 14.6 kyr BP based on a combination of different explanations. Best-guess simulation results of atmospheric Δ^14^C (blue) superimposed by a linear trend of either −0.04‰ per year (long dashed line) or −0.10‰ per year (solid line) (short dashed: no trend superimposed). (**b**) Atmospheric CO_2_. EDC ice core CO_2_ data (mean ±1*σ*) on two different chronologies^3^,^4^ AICC2012 and Parrenin2013. Simulated true atmospheric CO_2_ rise (black bold line), and how the signal might be recorded in EDC (dashed red line) after filtering for gas enclosure and shifted by 50 years to meet the data. (**c**) Simulated peak height in atmospheric Δ^14^C (grey areas) as function of length of carbon release and of the Δ^14^C depletion. (**d**) Simulated peak height in atmospheric CO_2_ (dark blue area) as function of length of carbon release. In **c**,**d**, simulations result with the AMOC in either a weak or a strong mode are combined spanning a range of results. Magenta square and circle in **c**,**d** mark results of our best-guess scenario for Δ^14^C and CO_2_, respectively. We colour coded the areas in the parameter space where simulation results agree with the EDC CO_2_ data (**d**, light blue) and with the interpretation of the Tahiti Δ^14^C data (**c**, black boxes). The latter are modified for background linear trends already contained in IntCal13 based on other processes.

**Figure 5:**
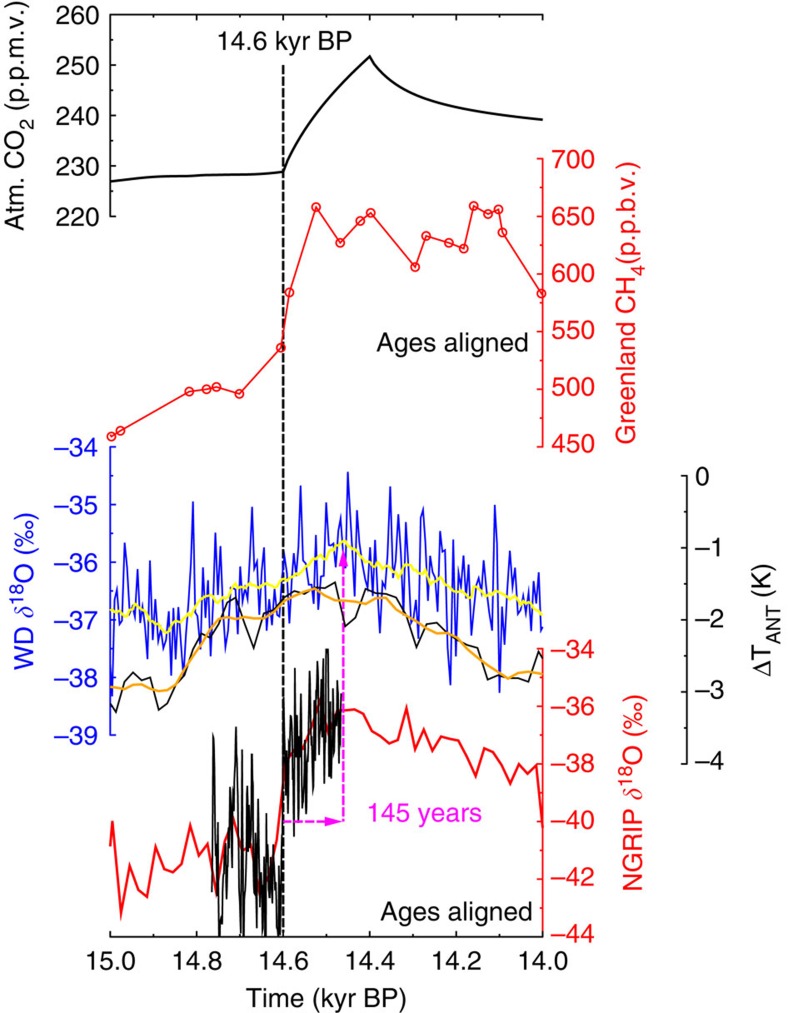
Implication on the timing of abrupt climate change as obtained in various ice core records from Greenland and Antarctica. Our results suggest that anomalies in Tahiti Δ^14^C and true atmospheric CO_2_ are caused by the same process. This information is used here as an independent age constraint. The onset of the abrupt rise in atmospheric CO_2_ (black bold, this study) is thus tied to 14.6 kyr BP. From previous ice core analysis^22^, it is known that the rise in CO_2_ and CH_4_ (red circles, Greenland composite^69^) occur synchronously here. A new study^21^ on the NEEM ice core tied the CH_4_ rise to be near synchronous to Greenland temperature rise. This synchronicity of the start of the abrupt changes in atmospheric CO_2_, CH_4_ and Greenland temperature tied to 14.6 kyr BP led to the age alignments in CH_4_ and NGRIP *δ*^18^O (high^24^ (black thin line) and low^70^ (red line) resolution). We furthermore show some Antarctic climate records on their own independent chronologies to illustrate the temporal north–south offsets. WD^54^ *δ*^18^O, original (blue) and 100 years running mean (yellow) and stack^3^ of temperature change from five ice cores in East Antarctica, Δ*T*_ANT_, original (black) and 100 years running mean (orange).

**Figure 6:**
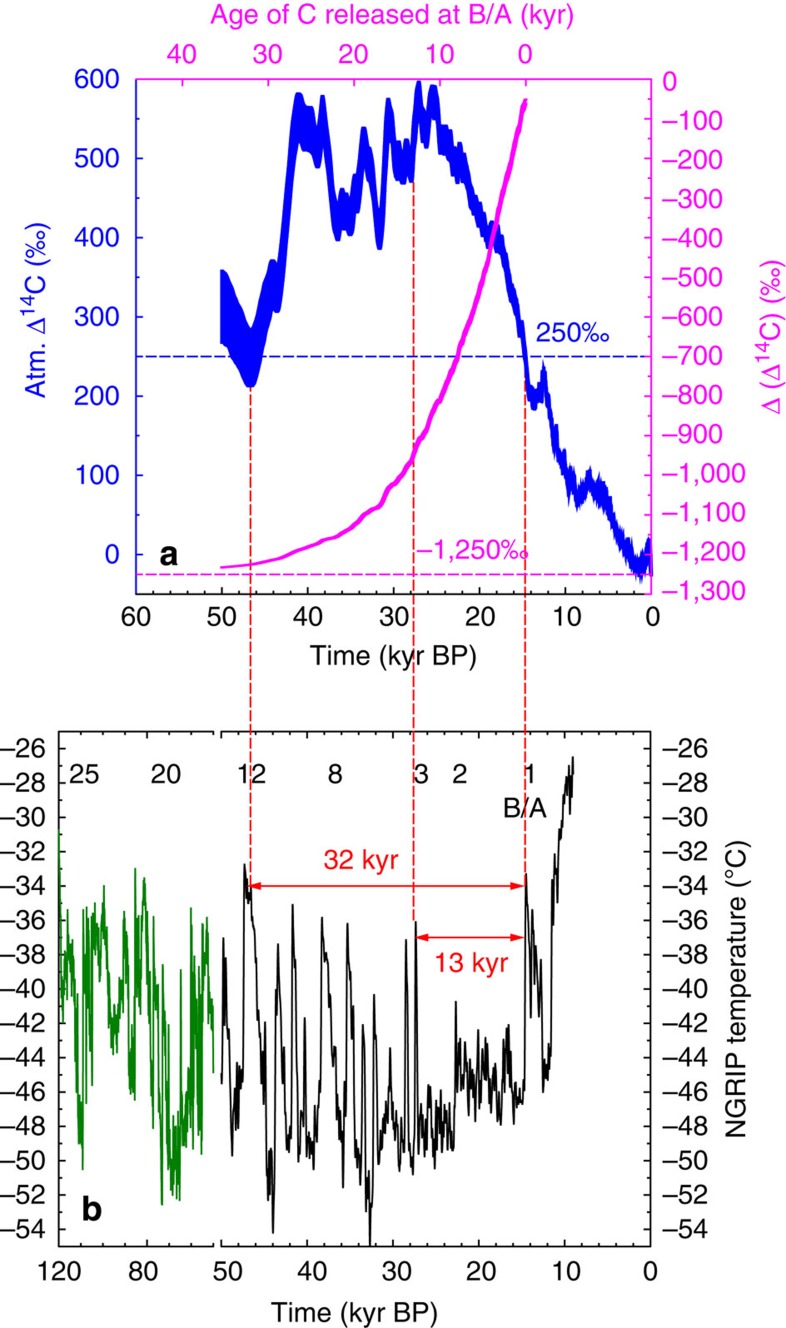
Radiocarbon depletion of soil carbon of different age and high northern latitude climate change. (**a**) Atmospheric Δ^14^C based on IntCal13 (ref. 9) over the last 50 kyr (blue, left *y* axis, mean ±1*σ*). Calculated radiocarbon depletion resulting in Δ(Δ^14^C) (mean ±1*σ*) of soil carbon released during the B/A as a function of its age (magenta, right *y* axis, upper *x* axis) and of atmospheric Δ^14^C during time of production. (**b**) NGRIP temperature reconstruction^33^ from 120 to 10 kyr BP. The time series is plotted in two different colours because of the break in the *x* axis scale at 50 kyr BP. Numbers label selected D/O events. Red labelled arrows highlight the time which past since NGRIP was similar as warm as during the B/A (32 kyr since D/O event 12) and since the previous significant warming before the B/A (13 kyr since D/O 3).

**Figure 7:**
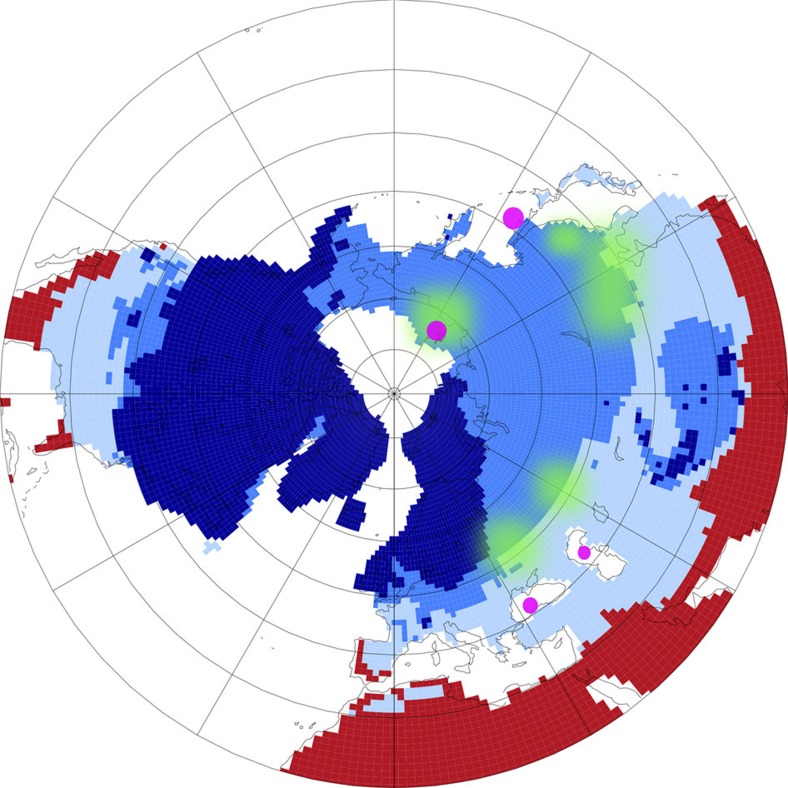
PMIP3 simulation results on the LGM permafrost extend. Results^41^ show a polar projection of the NH from 20 °N northwards, are based on soil temperature and distinguish land with ice (dark blue), permafrost (blue), seasonal frozen (light blue) and not frozen (red). Present day coastlines are sketched in thin black lines. Magenta points mark potential core sites (Siberian Shelf, Black Sea, Caspian Sea, Sea of Okhotsk) from which future ^14^C measurements on terrigenous material might verify the age of permafrost possible thawed around 14.6 kyr BP (suggested green areas).

We simulate the release of 125 Pg of carbon into the atmosphere with a constant rate that varies inversely with the time length of the event between 0.42 Pg C per year (300 years) and 2.5 Pg C per year (50 years) and configured the AMOC in either its strong or its weak mode. Both AMOC configurations differ in the strength of the overturning cell in the Atlantic with 16 Sv deep water production in the North Atlantic in the strong mode and 2 Sv in the weak mode. We repeated our previous comparison^6^ of simulated atmospheric *δ*^13^CO_2_ to ice core data from the EDC because new *δ*^13^CO_2_ data were published in the mean time^1^. For this model-data comparison of *δ*^13^CO_2_, we distinguished terrestrial and marine sources of the released carbon by assuming a *δ*^13^C signature of −22.5 and −8.5‰, respectively (Fig. 3b). More details on these assumptions are found in our previous article^6^. Δ(Δ^14^C) of the simulated carbon release is depleted with respect to the atmosphere between −50 and −1,250‰. Initial conditions of ^14^C production rates influence simulated Δ^14^C over several ten thousand years^32^. All simulations therefore start at 60 kyr BP. In our standard case, ^14^C production rates are assumed to be constant and 15% higher than present day, leading to atmospheric Δ^14^C of +250‰ at 14.6 kyr BP in agreement with IntCal13 (ref. 9). Long-term trends in ^14^C production rate as suggested by the geomagnetic field data^32^ only slightly impact our simulations (Supplementary Fig. 4).

For model evaluation, BICYCLE is (a) compared in its oceanic carbon uptake dynamic resulting in a model-specific airborne fraction with other models, (b) used to simulate the Suess effect (years 1820–1950 AD), (c) the bomb ^14^C peak (years 1950–2000 AD) and (d) applied on CO_2_ release experiments for preindustrial background conditions. The model is compared with the results from another carbon cycle box model^66^ (Suess effect and for preindustrial conditions) and with output from the GENIE model^67^, an Earth system model of intermediate complexity (preindustrial conditions). All details on this model evaluation are found in the Supplementary Note 2 including Supplementary Figs 6–8.

### Filtering true atmospheric CO_2_ into signals recorded in EDC

The smoothing effect of the gas enclosure process in ice cores that transforms a potential true atmospheric CO_2_ into a time series comparable to EDC ice core data is performed with a log-normal probability density function with an assumed mean value or width *E* of 400±80 years (mean ±1*σ*)^6^ (Supplementary Fig. 2c):


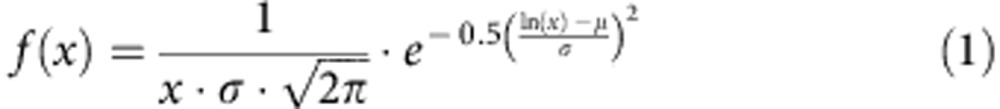


with *x* (in years) as the time elapsed since the last exchange with the atmosphere. From the two free parameters, *μ* and *σ* of the equation, we chose for simplicity *σ*=1, which leads to *E*=*e*^*μ*−0.5^. The application of such a filter function for the transformation of true atmospheric signals into those that might be recorded in ice cores during rapid climate change was compared with results from firn densification models and extensively validated with CH_4_ data from both hemisphere^6^.

## Author contributions

All authors designed research; P.K. performed carbon cycle simulations with BICYCLE; E.B. performed carbon cycle simulations with other box models; G.K. performed climate simulations; P.K. drafted the manuscript with contributions from all co-authors.

## Additional information

**How to cite this article:** Köhler, P. *et al.* Permafrost thawing as a possible source of abrupt carbon release at the onset of the Bølling/Allerød. *Nat. Commun.* 5:5520 doi: 10.1038/ncomms6520 (2014).

## Supplementary Material

Supplementary Information Supplementary Figures 1-8, Supplementary Notes 1-3 and Supplementary References
