# Supplementary material for: Permafrost thawing as a possible source of abrupt carbon release at the onset of the Bølling/Allerød
Source: Nat Commun. 2014 Nov 20;5:5520. doi: 10.1038/ncomms6520 (PMC4263146; doi:10.1038/ncomms6520)
Supplement: Supplementary Information — Supplementary Figures 1-8, Supplementary Notes 1-3 and Supplementary References [file ncomms6520-s1.pdf]

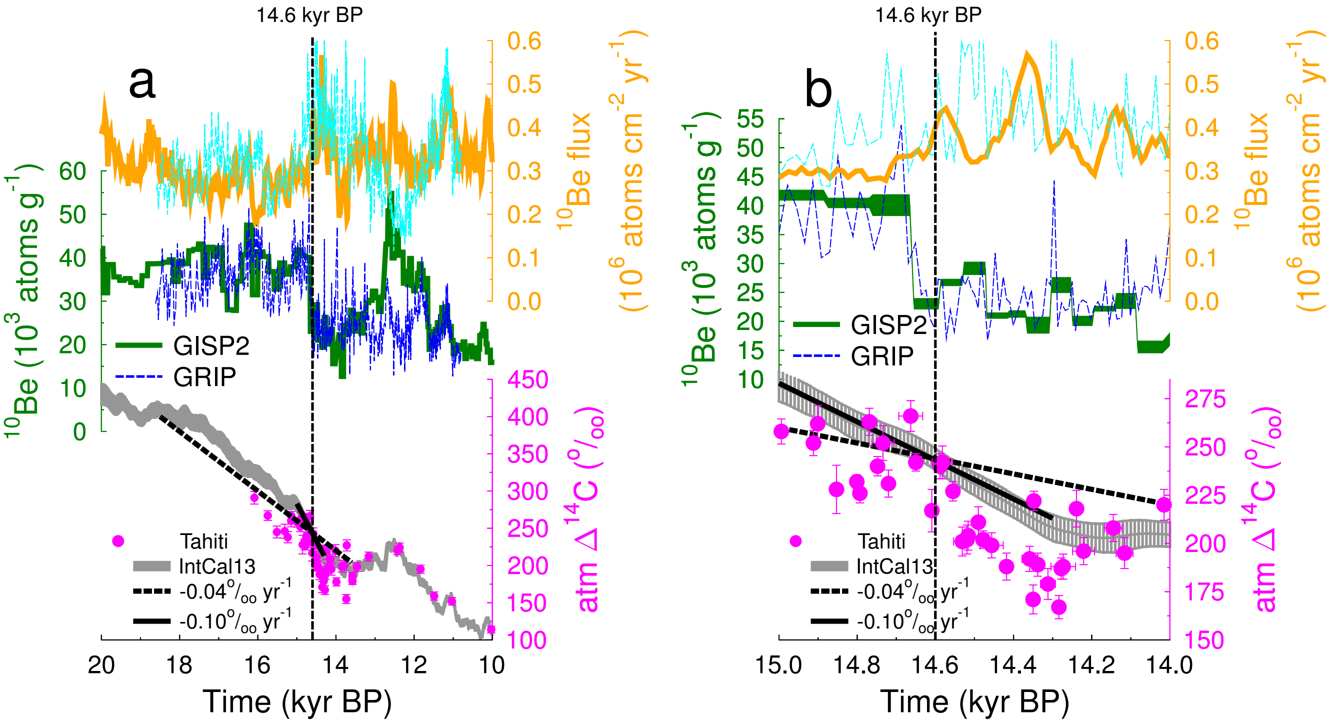

Supplementary Figure 1: Comparing atmospheric ^{10}Be and Δ^{14}C data to evaluate the potential impact of variable ^{14}C production rates on Δ^{14}C. (a) Termination I. (b) Focus on 15 to 14 kyr BP. ^{10}Be fluxes and concentration of Greenland ice cores GISP2[1] (bold closed lines, green and orange) and GRIP[2] (dashed lines, blue and cyan) on GICC05[3] age model. Atmospheric Δ^{14}C from IntCal13[4] (grey band of $\pm 1\sigma$ around the mean) and Tahiti corals[5] (magenta, $\pm 1\sigma$ in both age and Δ^{14}C) including a linear trend with -0.04‰ yr^{-1} or -0.10‰ yr^{-1}.

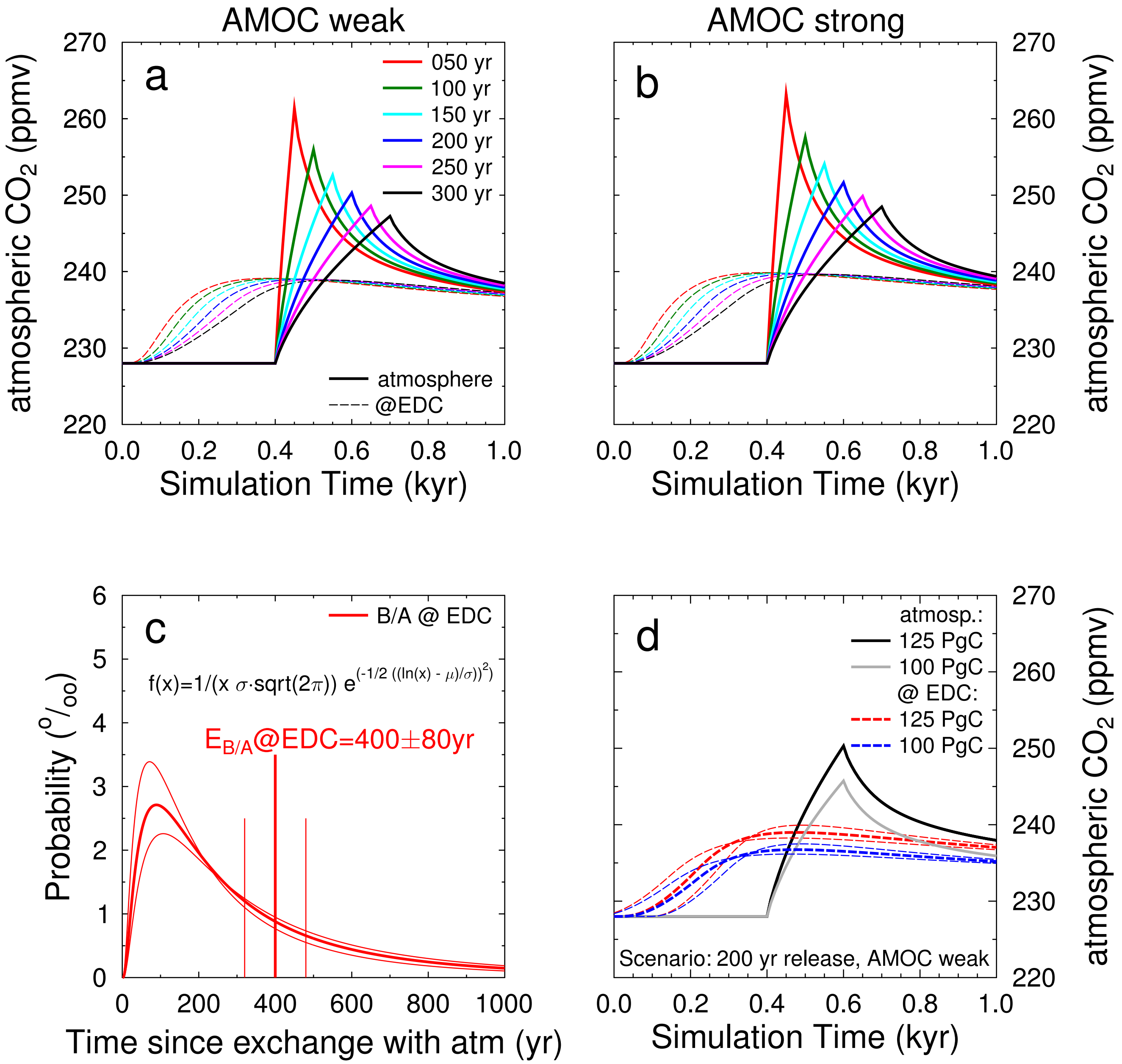

Supplementary Figure 2: Simulated changes in atmospheric CO_2 using the BICYCLE carbon cycle model. The model is set-up for around 14.6 kyr BP. Simulations vary in the length of the carbon release between 50 yr and 300 yr, AMOC is (a) weak or (b) strong. Both the atmospheric signal (bold) and the smoothed signal (dashed) that would be recorded in EDC are plotted. No age correction during smoothing is applied. (c) The atmospheric time series are filtered (smoothed) to address the firn air mixing before gas enclosure in ice cores with a log-normal probability density (PDF) function, that was tested with output of firn densification models[6]. The mean width E of PDF corresponds to the climatic conditions around 14.6 kyr BP at EDC ($E = 400 \pm 80$ years (1σ)). The PDF follows Equation 1 shown in the methods (main text), in which μ was chosen in order to follow $E = e^{\mu - 0.5}$ for the given mean width $E = 400$ years (bold lines) or 320 and 480 year, respectively (thin lines). (d) Comparing how the true atmospheric CO_2 signal in two scenarios (125 or 100 PgC released in 200 years, AMOC off) might be recorded in EDC including the 1σ uncertainty in the log-normal PDF (subfigure c) used for smoothing during gas enclosure.

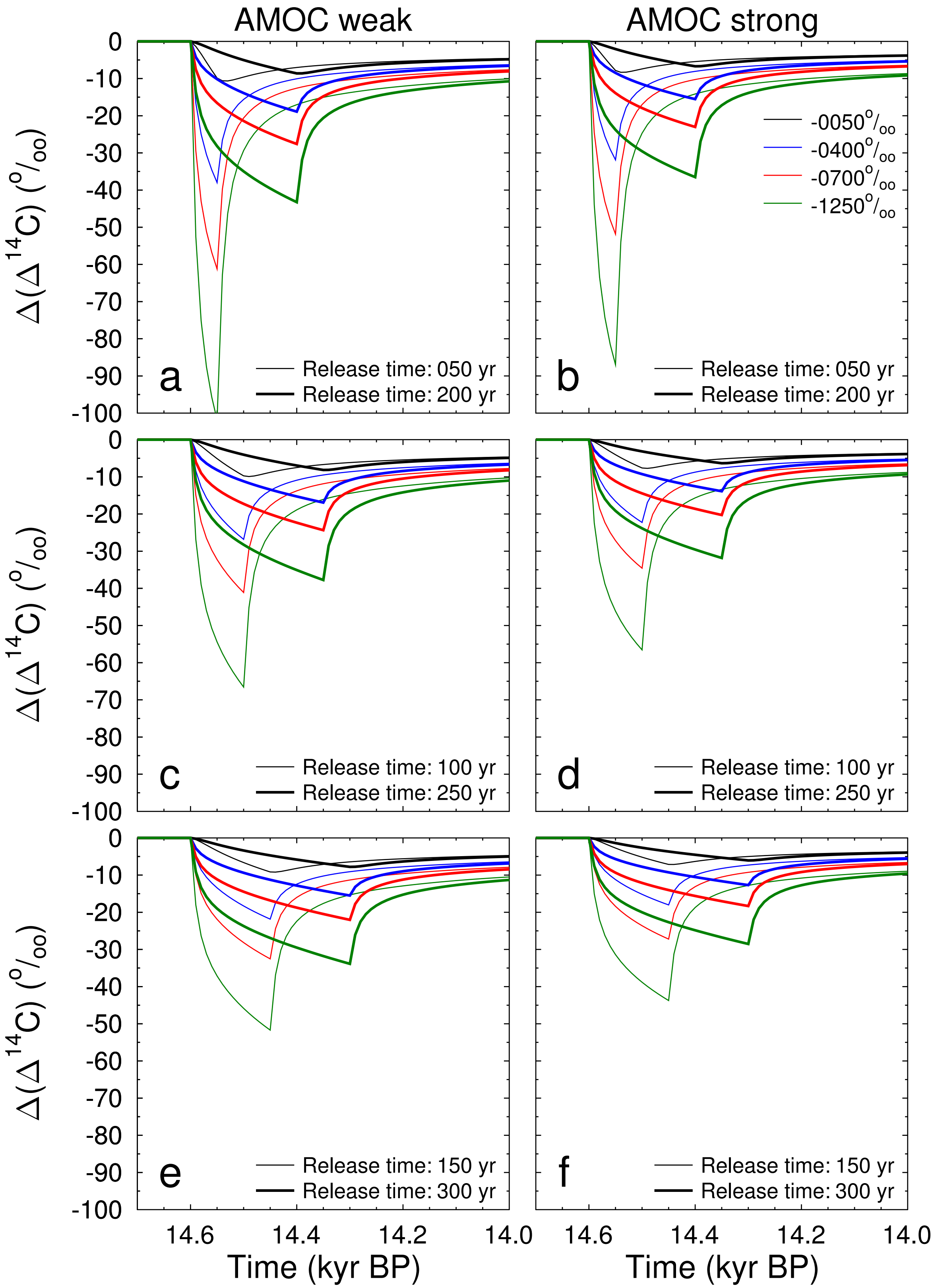

Supplementary Figure 3: Simulated changes in atmospheric Δ^{14}C using the BICYCLE carbon cycle model. The model is set-up for around 14.6 kyr BP. Simulations vary in the length of the carbon release: a,b) 50 and 200 years; c,d) 100 and 250 years; e,f) 150 and and 300 years. AMOC is weak (left) or strong (right), and ^{14}C depletion of released carbon with respect to atmosphere varying between –50‰ and –1250‰. ^{14}C production rate was constant at $1.15\times$ pre-industrial level, which gives after 45 kyr of spin-up time an atmospheric Δ^{14}C of about 250‰, comparable with the observations.

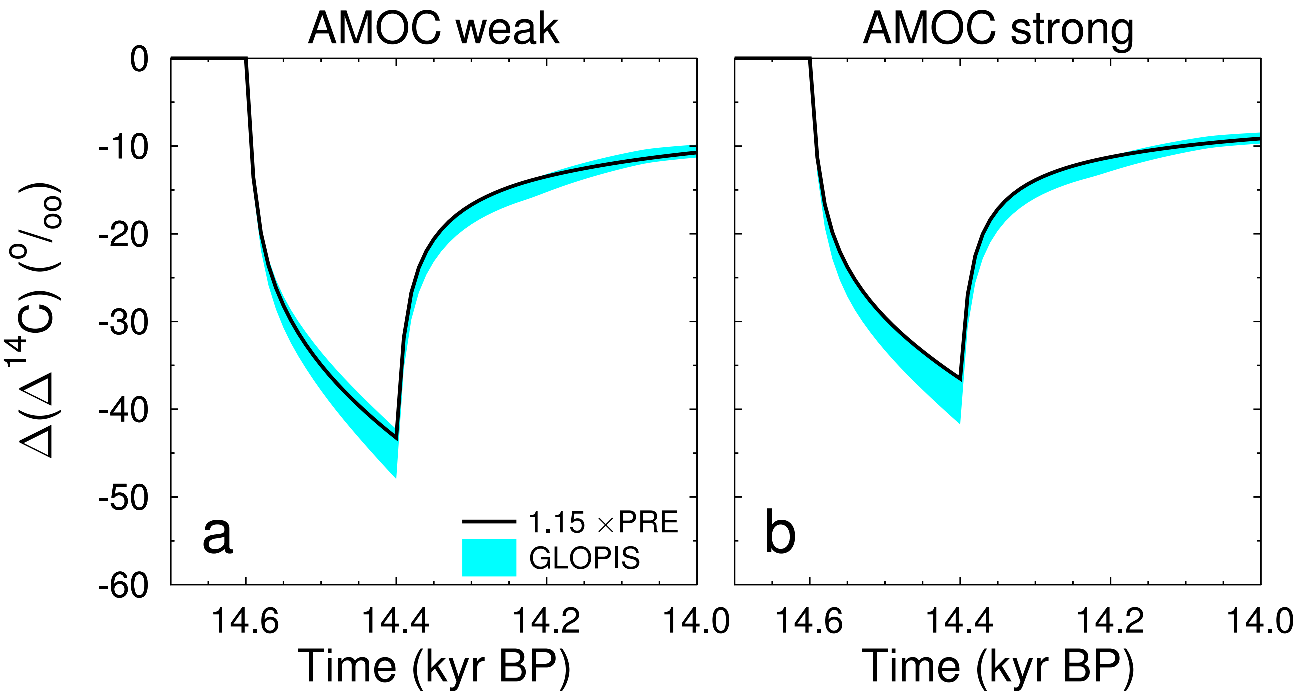

Supplementary Figure 4: Impact of long-term change in ^{14}C production rate on simulated atmospheric Δ^{14}C. The ^{14}C production rate were derived from the geomagnetic field (GLOPIS) and applied in the BICYCLE model as in a previous application[7]. Results for our best guess scenario (-1250‰, 125 Pg carbon released in 200 years) with 45 kyr of spin-up time are shown. AMOC is in (a) weak or (b) strong mode. Cyan band spans results based on either a minimum or a maximum change in ^{14}C production rate as deduced from GLOPIS[7].

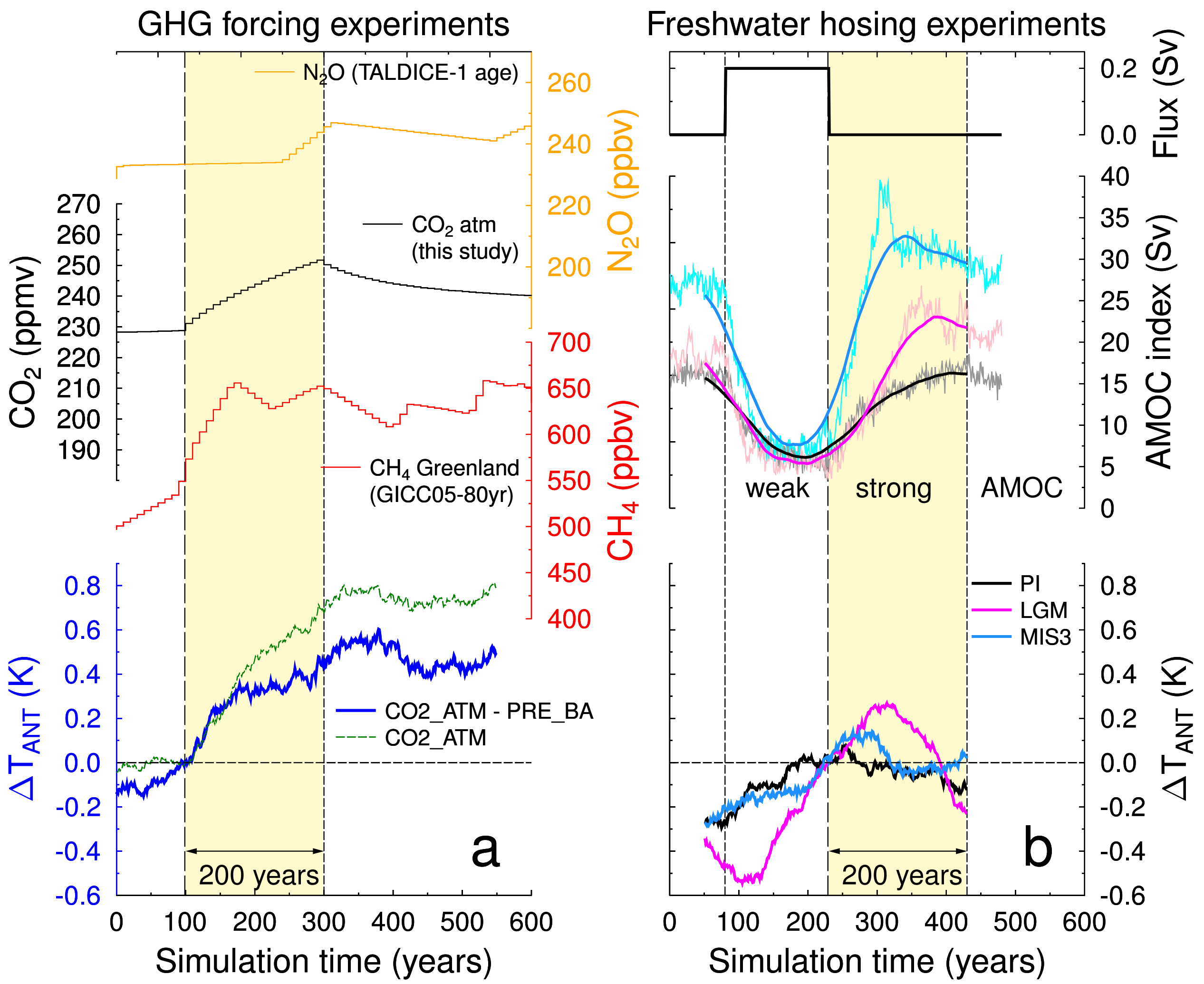

Supplementary Figure 5: Results of transient climate model simulations using COSMOS. Shown are the simulated annual mean Antarctic surface temperature change ΔT_{ANT} in (a) our GHG forcing experiments and (b) a reanalysis of North Atlantic freshwater hosing experiments[8,9], which serve as a surrogate for the simulation of abrupt climate changes. Recently it has been shown that the recovery and amplification of the AMOC at the end of freshwater perturbations is strongly dependent on the climate background state[8]. Therefore we analysed the Antarctic temperature response for three different climate states (PI: pre-industrial; LGM: Last Glacial Maximum at 21 kyr BP; MIS3: 32 kyr BP) that cover a wide spectrum of glacial-interglacial conditions to evaluate the robustness of the Antarctic temperature response. In Panel (a) we show the GHG N_2O, CO_2 and CH_4 as used for forcing the model in simulation CO2_ATM and the simulated anomaly in ΔT_{ANT} in scenario CO2_ATM (green dashed) and of CO2_ATM–PRE_BA (blue), both as 100 years running averages with respect to year 100. Vertical lines bracket the rise in atmospheric CO_2, representing 14.6 and 14.4 kyr BP, respectively. In panel (b) the North Atlantic freshwater hosing is shown in Sv (1 Sv = 10^6 m^3 s^{-1}). The freshwater perturbation with a freshwater flux of 0.2 Sv is added to the ice-rafted debris belt in the North Atlantic Ocean, around 40°N – 55°N, 45°W – 20°W of the central Atlantic Ocean. The intensity of the AMOC is presented by the maximum value of the meridional overturning stream function of the upper 200–3000 m and 30°N northward[8]. The simulated anomaly in ΔT_{ANT} for the different background climate states are shown as 100 years running averages with respect to year 230 (i.e. the end of the freshwater perturbation). Hence the corresponding key intervals marked in apricot background colour in panel a) and b) are between 100-300 years and 230-430, respectively.

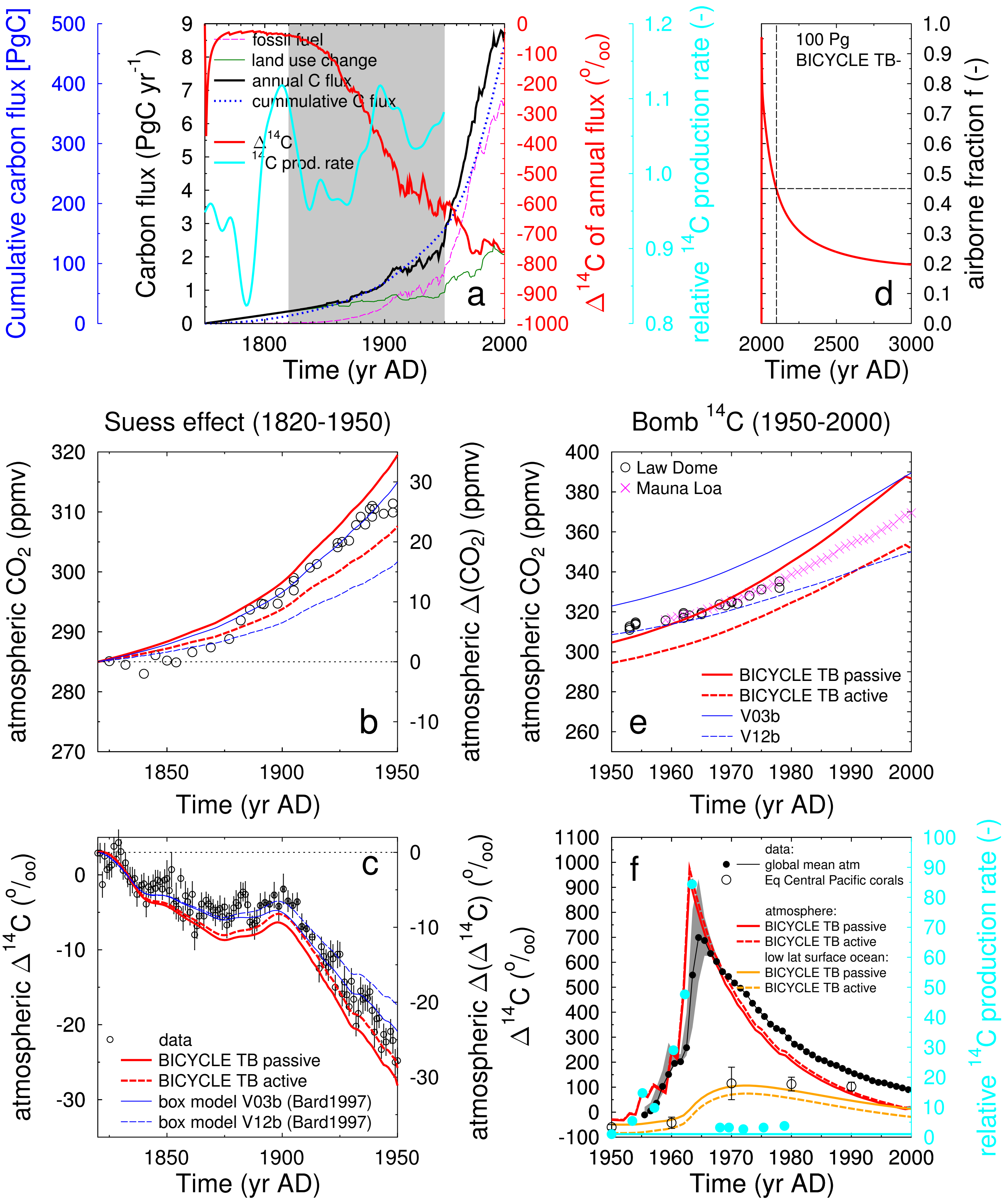

Supplementary Figure 6: Evaluation of ^{14}C model performance for Suess effect and bomb ^{14}C peak. See caption on the following page.

Supplementary Figure 6: Evaluation of ^{14}C model performance for Suess effect and bomb ^{14}C peak. (a) Fluxes of fossil fuel emissions[10] (magenta), and land use change[11] (green, between 1850 AD and 1750 AD linearly extrapolated to zero). Assumption for Δ^{14}C signature of total anthropogenic C flux to the atmosphere (red): Land use change extracts woody parts of trees (fractionated by $-40‰$ during photosynthesis from atmosphere, no further ageing), fossil fuels are ^{14}C-free (Δ^{14}C$= -1000‰$). Relative changes in ^{14}C production rate (cyan) with respect to the long-term Holocene[12]. Gray box (1820–1950 AD) covers bomb-free Suess effect data[13]. (b) Simulated changes in atmospheric CO_2 against CO_2 data from the Law Dome ice core[14] (circles). BICYCLE model (red) with terrestrial biosphere (TB) either passive (constant, solid lines) or active (changes as function of CO_2, dashed lines). A second box model in two different versions (blue) for comparison[15]. (c) Simulated changes in atmospheric Δ^{14}C against reconstructions (circles with error bars (1σ)[13]). BICYCLE started from 10 kyr BP, the 12-box model started at 0 AD, both used the same relative ^{14}C production rate changes[12]. (d) Airborne fraction f over time for the injection of 100 PgC in year 2001 (background CO_2 of 389 ppmv) in BICYCLE with terrestrial biosphere passive (TB-). No fossils fuel emission after year 2000. f is calculated from the difference to simulation without 100 PgC injection for the next 1000 years. Dotted lines mark f after 100 years. (e) Simulated atmospheric CO_2 in the bomb ^{14}C-peak time window (1950–2000 AD) against CO_2 data (Law Dome[14], Mauno Loa CO_2 annual mean[16]). (f) Simulated bomb ^{14}C-peak against reconstructions. Assumed additional bomb-related ^{14}C production rate calculated after Naegler[17], but normalised to the mean natural ^{14}C production rate used here (85% of Naegler[17]) leading to the input of $1.2 \cdot 10^6$g ^{14}C into the atmosphere. Atmospheric ^{14}C data[18] (global mean (black filled circles) with range measured in high latitude northern hemisphere and southern hemisphere (grey area) and coral-based reconstructions of central equatorial Pacific surface waters[19] (open circles with error bars denoting the observed range) against simulations with BICYCLE.

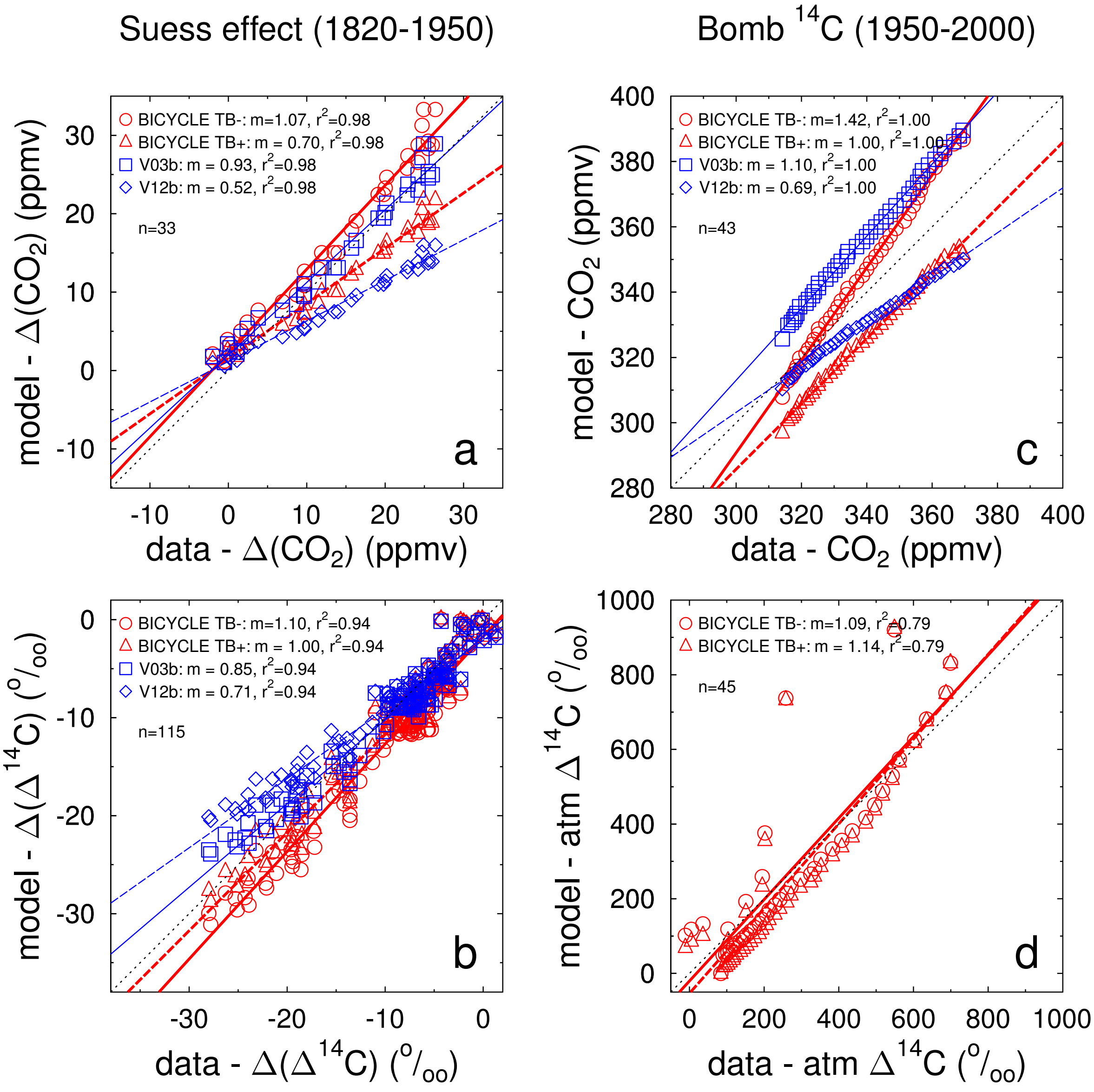

Supplementary Figure 7: Suess effect and bomb ^{14}C peak. Scatter plots of model-data comparison for changes in atmospheric CO_2 and $\Delta^{14}C$ for the Suess effect and the bomb ^{14}C peak. (a) Atmospheric CO_2 for the Suess effect. (b) Atmospheric $\Delta^{14}C$ for the Suess effect. (c) Atmospheric CO_2 for the bomb ^{14}C peak. (d) Atmospheric $\Delta^{14}C$ for the bomb ^{14}C peak. Model results are resampled only for the n years in which data points are available. The dotted black lines denotes the perfect model-data agreement (slope $m = 1.0$). The calculated slopes m and the correlation coefficients r^2 of the linear regressions are contained in the legends.

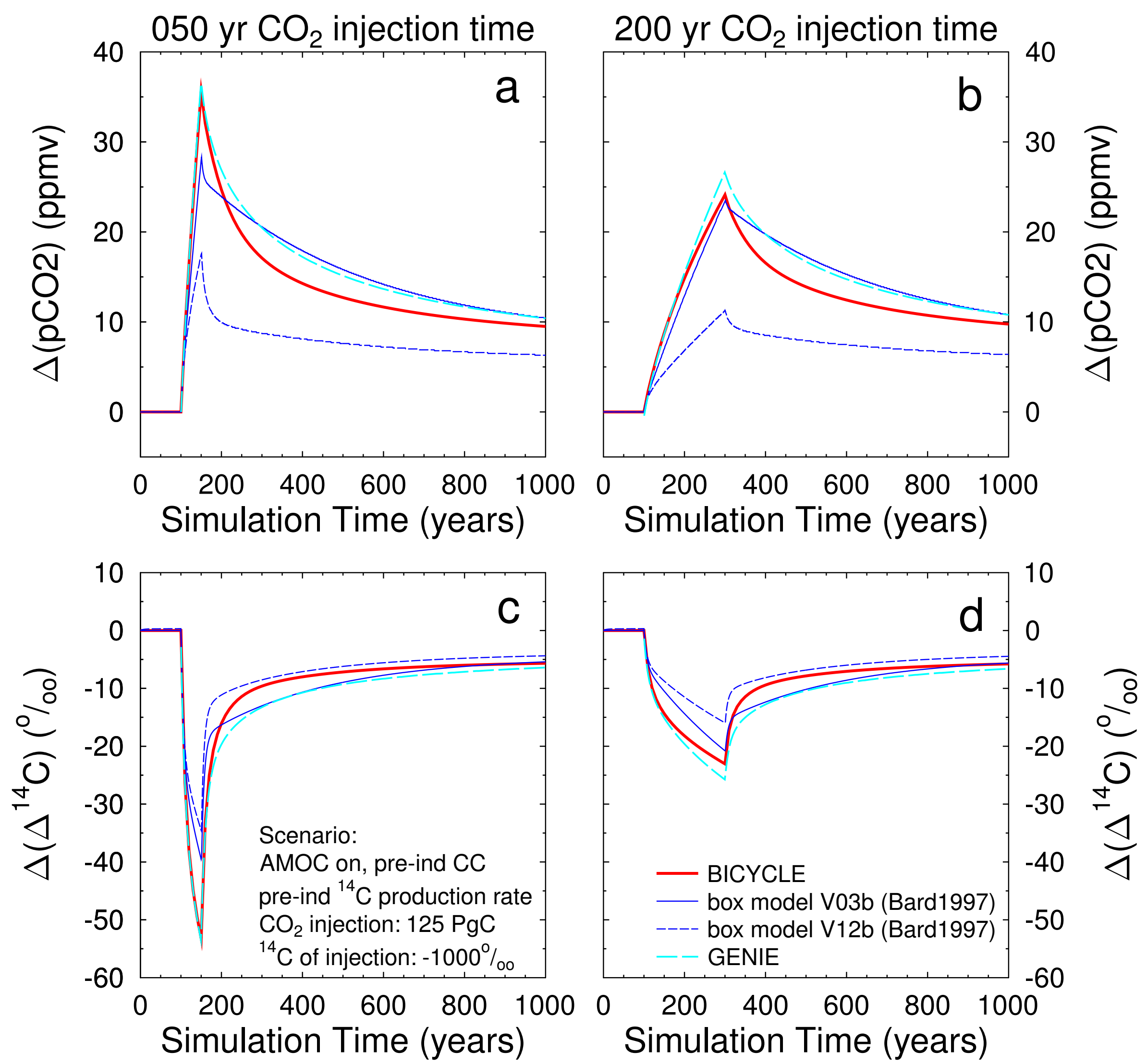

Supplementary Figure 8: Comparing different carbon cycle models. BICYCLE (red), the other box model[15] (blue, two versions), and the Earth System model of intermediate complexity GENIE[20] (cyan) are compared for a pre-industrial CO_2 injection scenario (AMOC strong, pre-industrial carbon cycle, pre-industrial ^{14}C production rate) for 125 PgC injection with ^{14}C signature of $-1000‰$. Injection started in simulation year 100 for a length of 50 (left) or 200 (right) years. Top: Changes in atmospheric CO_2, bottom: changes in atmospheric $\Delta^{14}C$.

Supplementary Note 1

Atmospheric Δ^{14}C Data. Atmospheric Δ^{14}C deduced from corals might be influenced by temporal changes in the reservoir ages, or by variable ^{14}C production rates. Both potential sources of uncertainty are briefly discussed below:

(a) Reservoir ages

Reservoir ages might vary with time due to changes in (i) ocean circulation or (ii) gas exchange rates[21]:

(i) Especially around the end of Heinrich Stadial 1 the resumption of the Atlantic meridional overturning circulation (AMOC) might lead to variable reservoir ages. This leads, for example, for Atlantic sites in the Cariaco Basin in an ocean carbon cycle model to uncertainties and changes in the reservoir age of the order of 100 years within a few centuries[22]. This time-dependent change in reservoir age leads to a difference in atmospheric Δ^{14}C of less than 5‰ if compared to results based on a constant reservoir age. The influence of the AMOC resumption around 14.6 kyr BP on reservoir ages should be smaller in the central Pacific around Tahiti than in the Caribbean around Cariaco. Another simulation study[23] focusing on the impact of AMOC changes during the Younger Dryas also found rather constant reservoir ages throughout most of the Pacific. Furthermore, wind-driven circulation or ventilation might also impact on the reservoir age. However, as discussed in the next paragraph in detail we do not find any indication that surface wind speed around Tahiti was changing, it stayed remarkable stable in all simulation results which were available to us covering stable climates for modern times and LGM, and abrupt climate changes during MIS 3 and the last deglaciation (this study and [8,9]). Reservoir age changes based on ocean circulation change is therefore very small and thus negligible.

(ii) Gas exchange rates of CO_2 between atmosphere and surface ocean depend mainly on local wind speed. In existing simulations local surface wind speed around Tahiti (about 150°W, 18°S) are remarkable stable. In simulations with the COSMOS coupled Earth system model (see further below) surface wind speed around Tahiti never changes by more than 10% from its pre-industrial values. This is the case in simulation results for LGM climate[9], for our simulation scenarios PRE_BA and CO2_ATM (this study), or across an AMOC shutdown and resumption[8]. We therefore found no supporting evidence for a wind speed change in Tahiti across an abrupt stadial/interstadial transition comparable to the B/A around 14.6 kyr BP and therefore assume that the impact of reservoir age change on the interpretation of the coral-based Δ^{14}C data from Tahiti was small and negligible.

A recent study[24] used the same Δ^{14}C data from Tahiti corals to come to a different interpretation of the reservoir age. They assumed that the IntCal09 stack provides the true atmospheric Δ^{14}C signal, which implied that all differences in Tahiti Δ^{14}C to IntCal09 has to be explained by a variable reservoir age R. Consequently, they found R to vary up to 400 years at the onset of the B/A. This is the opposite to our interpretation. Here, we rely on the Tahiti corals to be the most reliable recorder of Δ^{14}C — because of its small measurement uncertainty and precise U/Th dating. One main concern about the work by Kubota et al.[24] is the usage of the old IntCal09 curve rather than the new updated version[4] IntCal13. Indeed, the main difference between the two IntCal curves occurs around the Heinrich 1 event with significant improvements included in the IntCal13 curve. This can be briefly illustrated with a simple example of an age within the Heinrich 1 event: 16,500 calendar years BP corresponds to 13,310 ^{14}C years BP in the IntCal09 curve, but

13,682 ^{14}C years BP in the new IntCal13 curve. Consequently, the use of IntCal13 instead of IntCal09 reduces automatically by 372 years the apparent ^{14}C reservoir age for a coral sample of that calendar age. This is the magnitude of the change in ΔR that is interpreted erroneously by Kubota et al.[24] (cf. their Fig. 4 and related text) as due to CO_2 release by the ocean.

(b) Changes in ^{14}C production rate

Atmospheric Δ^{14}C might also vary because of a change in ^{14}C production rate, which would also be visible in other cosmogenic isotopes, e.g. in the atmospheric concentration of ^{10}Be which is available from the GISP2 ice core[1] and in high resolution from the GRIP ice core[2]. GISP2 ^{10}Be data on the GICC05 chronology[3] show a decrease in ^{10}Be concentration by 50% around 14.65 kyr BP, but a gradual rise in the ^{10}Be flux (Supplementary Fig. 1). Depending on the kind of deposition (wet, dry) atmospheric ^{10}Be concentration is more related to either ^{10}Be concentration or ^{10}Be flux in ice cores. The patterns found in ^{10}Be data in the GISP2 ice core are reproduced in higher resolution in the data from the GRIP ice core with some rapid overprints, mainly in the ^{10}Be flux data. Although during rapid climate changes it is not clear which deposition is dominant[1], neither of both ^{10}Be concentration or ^{10}Be flux shows a pronounced $\sim 200 - 250$ years long anomaly comparable with the new Δ^{14}C data making a ^{14}C production rate change a very unlikely explaination (see also[25] for a discussion of difficulties linked to wet and dry deposition of ^{10}Be). Furthermore, upcoming new ^{10}Be data from the WAIS Divide Ice Core, Antarctica, show no abrupt changes around 14.6 kyr BP, excluding changes in ^{14}C production rate as a plausible cause for the Tahiti Δ^{14}C anomaly[26,27].

Supplementary Note 2

Evaluation of the Carbon Cycle Model. For model evaluation BICYCLE is (a) compared in its oceanic carbon uptake dynamic resulting in a model-specific airborne fraction with other models, (b) used to simulate the Suess effect (years $1820 - 1950$ AD), c) the bomb ^{14}C peak (years $1950 - 2000$ AD), and (d) applied on CO_2 release experiments for pre-industrial background conditions. The model is compared with results from another carbon cycle box model[15] (Suess effect and for pre-industrial conditions) and with output from the GENIE model[20], an Earth system model of intermediate complexity (pre-industrial conditions).

(a) Airborne fraction

A recent model inter-comparison[28] with 14 models (three comprehensive Earth System Models, seven Earth System Models of Intermediate Complexity, and four box-type models) calculated the airborne fraction f of an instantaneous CO_2 emission of 100 PgC to a background atmospheric CO_2 of 390 ppmv. We here repeat this scenario[28] by adding 100 PgC in year 2000 of our anthropogenic emission scenario (BICYCLE, terrestrial biosphere passive) having then a CO_2 of 389 ppmv. After year 2000 no further anthropogenic emissions are considered. The airborne fraction f is then calculated from the differences in atmospheric CO_2 for a run without this 100 PgC release (Supplementary Fig. 6d). f declines towards 0.45 after 100 years and to 0.20 after 1000 years, thus slightly larger than the rough estimate of $f = 0.17$ after 1000 years given previously[6], in which the experiment and background conditions were different. These results agree well with the model-mean of the model inter-comparison[28], which obtained $f = 0.41 \pm 0.13$ (2σ) and $f = 0.25 \pm 0.09$ after 100 and 1000 years, respectively. A smaller airborne fraction (faster oceanic uptake

rate) would allow a larger amount of carbon released to the atmosphere to be confined with ice core data.

(b) Suess effect

Further evaluation of the ^{14}C dynamics in BICYCLE was obtained from simulating the Suess effect[13,29]: the impact of anthropogenic fossil fuel and land use change emissions[10,11] on atmospheric $\Delta^{14}C$ (Supplementary Fig. 6a-c). This evaluation was chosen, because the Suess effect contains a well recognised influence of carbon emissions on atmospheric $\Delta^{14}C$, which are of similar magnitude and duration as the carbon release we proposed for the onset of the B/A. We here apply the anthropogenic emission rates and use the BICYCLE model setup as previously published[30,31].

The pre-bomb $\Delta^{14}C$ data reconstructed from well dated tree-rings including the Suess effect[13] cover the years 1820–1950 with atmospheric $\Delta^{14}C$ decreasing from $+3‰$ to $-25‰$ (Supplementary Fig. 6c). In the original study[13] the $\Delta^{14}C$ data continue until the year 1954, however the reconstruction of the ^{14}C production rate[12] used for forcing the model stops at 1950, we therefore restrict our analysis also to results up to the year 1950. The $\Delta^{14}C$ data show especially a decline of $-23‰$ between 1900 and 1950, furthermore some multi-decadal feature before 1900. These atmospheric $\Delta^{14}C$ data contain the combined influence of the anthropogenic emissions and variations in the ^{14}C production rate on atmospheric $\Delta^{14}C$. The cumulative anthropogenic carbon emissions during 1820–1950 sum up to 145 Pg of C (Supplementary Fig. 6a). Their ^{14}C signature depends on the relative share of ^{14}C-free fossil fuel emissions and land use change. For the latter we assume a $\Delta^{14}C$ signature of $-40‰$, being a mean value of pre-industrial terrestrial carbon within BICYCLE. Anthropogenic emissions were dominated until the beginning of the 20th century by land use change[11] with annual output of fossil fuels being only of secondary importance[10,32]. The assumed mean $\Delta^{14}C$ of the total anthropogenic emissions before 1850 was therefore only -40 to $-100‰$, but decreased to $-600‰$ around 1950 and $-750‰$ around 2000 (Supplementary Fig. 6a). We use the recent calculation of ^{14}C production rates based on changes in the geomagnetic field strength and the solar activity[12]. These relative ^{14}C production rates changes (calculated with respect to the long-term Holocene mean) varies in the two century from 1750 to 1950 between 82% and 112% (Supplementary Fig. 6a).

Most important features of the observed changes in atmospheric CO_2 rise and $\Delta^{14}C$ decline are met by our simulations. We focus on simulated changes, not absolute values, therefore all model simulations shown in Supplementary Figs. 6b,c are shown as anomalies to year 1820 (right y-axes). Absolute values of simulations results differed from reconstructions by less than 15 ppmv (CO_2) and less than $5‰$ ($\Delta^{14}C$).

Atmospheric CO_2 (Supplementary Fig. 6b) measured in the Law Dome ice core rises by 25 ppmv between 1820 and 1950. Results with BICYCLE find a 22 ppmv rise in the model version with active terrestrial biosphere for the same time period, implying that photosynthesis on land is via the fertilisation effect a function of atmospheric CO_2. If terrestrial carbon content is kept constant (passive terrestrial biosphere) the simulated rise in atmospheric CO_2 between 1820 and 1950 is 35 ppmv, 10 ppmv higher than in the ice core data.

Changes in atmospheric $\Delta^{14}C$ (Supplementary Fig. 6c) include both the rather steep decline after the year 1900, but also important multi-decadal features of the $\Delta^{14}C$ data consisting of a local maximum in $\Delta^{14}C$ around 1900, a declining trend before 1840 and rather stable values in-between. Those features are caused by corresponding anomalies in the ^{14}C production rates (Supplementary Fig. 6a). In the BICYCLE

simulations the steep declines in the first two decades and after year 1900 are met well, in the intermediate period of rather stable Δ^{14}C, the model produces an offset with respect to the data of $3-5$‰. The difference in the two model applications with either active or passive terrestrial carbon cycle have only minor influence of ~ 2‰ on the simulated changes in atmospheric Δ^{14}C. The model version with active terrestrial biosphere, which also compares best with changes in atmospheric CO_2, produces a decrease in atmospheric Δ^{14}C of -28‰ between 1820 and 1950 for the Suess effect (including natural variations in the ^{14}C production rates) which is in very good agreement with the data.

The overall model-data misfit for the time window $1820-1950$ is estimated from linear regression analysis of simulated changes against observated changes (Supplementary Fig. 7a,b). Here, only simulation results are picked for the n years in which observational data are available. An optimal model would have in such a scatter plot all points on the diagonal with slope $m=1.0$. We here find for the simulated atmospheric CO_2 compared to the $n=33$ data points of Law Dome regression coefficients m of the slopes of 1.07 and 0.70 BICYCLE with passive and active terrestrial biosphere, respectively. For changes in atmospheric Δ^{14}C ($n=115$) the regression coefficients m of the slope are 1.10 and 1.00, listed again for the same simulation scenarios. These linear regression analyses led furthermore to $r^2 \geq 0.94$ for all model-data comparsions.

(c) Bomb ^{14}C peak

After the year 1950 the natural ^{14}C signals is overprinted by anthropogenic ^{14}C production during nuclear bomb testing. The so-called bomb ^{14}C peak will be used as an additional model evalution. The magnitude of the Δ^{14}C anomaly was by about a factor of 20 larger and faster than our carbon cycle anomaly during the onset of the B/A, but the data are still of use for analysing model dynamics.

Anthropogenic carbon emission in the setup for the Suess effect were already extended until year 2000 (Supplementary Fig. 6a). To investigate the bomb ^{14}C peak we need as additional forcing the bomb-induced artificial ^{14}C production. The background ^{14}C production rate varied before 1950 by 20% around the mean Holocene values (of here 440 mol yr^{-1}). From results of a ^{14}C budget closure[17] we calculate that the relative ^{14}C production rate increased in selected years (cyan points in Supplementary Fig. 6f). In the peak years $1960-1963$ this increase was by a factor of $30-84$. The cumulative additional ^{14}C production leads to the injection of $1.2 \cdot 10^6$g ^{14}C into the atmosphere after 1950. This is 15% smaller than intially[17] suggested, because our natural background ^{14}C production rate is only 85% of that chosen by Naegler[17].

The anthropogenic carbon emissions lead in the Law Dome[14] and Mauno Loa[16] data to a rise in CO_2 of 60 ppmv, from ~310 ppmv in year 1950 to nearly 370 ppmv fifty years later (Supplementary Fig. 6e). The simulated CO_2 rise in BICYCLE (terrestrial biosphere active) is identical (slope $m=1.0$ in data-model scatter plot (Supplementary Fig. 7c)), but offset by -15 ppmv. With passive terrestrial biosphere atmospheric CO_2 in BICYCLE rises faster leading to an amplitude of 85 ppmv, or a slope $m=1.42$ in the data-model scatter plot.

The bomb ^{14}C peak was observed in the atmosphere at various station around the globe, and shows a distinct north-south gradient before the year 1970. The calculation of a global mean ^{14}C peak is therefore not straightforward. We here show a previously[18] calculated mean, but also the range of the data between high northern latitude and southern hemisphere (Supplementary Fig. 6f). The global mean in atmospheric Δ^{14}C peaks in the data in the mid 1960s at 700 ± 200‰, and declines towards $+100$‰ in year 2000

thereafter. Additional information can be gained from the rise in Δ^{14}C in corals, recording surface ocean signals. From a coral-data compilation[19] we show (Supplementary Fig. 6f) the range of results obtained in central equatorial Pacific surface waters, because these waters are less perturbed by upwelling or ocean gyres, and can be used for comparison with our simple carbon cycle box model. These coral data record the bomb ^{14}C peak by a rise from $-50‰$ in year 1950 towards $+100‰$ from year 1970 onwards.

BICYCLE simulates the bomb ^{14}C peak by an atmospheric Δ^{14}C of $+1000‰$ in year 1963, and a decay towards $0‰$ in year 2000. Results differ only slightly for terrestrial biosphere either active or passive. The model-data scatter plot (Supplementary Fig. 7d) shows a slope m of 1.09 and 1.14, and $r^2 = 0.79$ for both BICYCLE realisations. Simulated surface equatorial Pacific Δ^{14}C nicely shows the rise from 1950 to 1970 by +150‰ similar to the coral data, but simulations decline thereafter faster than in the data. The decay of the Δ^{14}C peak in atmosphere and surface ocean that is faster in the model than in the data indicates that the vertical mixing between surface and deep ocean in the model operates faster than in nature. This is a phenomenom well known for box models (in detail, the mixing in the high latitude is faster in box models than for GCMs, see the calculation of the Harvarton-Bear index[33,34], from which we quantified that this effect is less pronounced in BICYCLE than in other box models[34]). The effect is the more pronounced the larger the gradient in the tracers between surface and deep ocean is. The bomb ^{14}C peak is therefore only of limited usage for the evalution of the model dynamics of BICYCLE, when we want to apply the model to the B/A event, a problem with more than an order of magnitude smaller changes in the ^{14}C cycle.

(d) Carbon cycle model comparison

To obtain further knowledge on how the carbon cycle model performs and on how simulations depend on the specific chosen model setup and parametrization we repeat the Suess effect experiments with another carbon cycle box model[15] (Supplementary Fig. 6). Furthermore, CO_2 release experiments, similar to the release proposed by permafrost thawing around 14.6 kyr BP, but with pre-industrial background conditions are compared for both carbon cycle models, but also with output from the more complex GENIE model (Supplementary Fig. 8). This simplified setup is used as a sensitivity test under generic and comparable conditions.

The other box models have been used previously to study the influence of ^{14}C production changes[15] and thermohaline circulation changes[35]. Two geometries have been used with a 12 box model and a simpler 3 box model (V12b & V3b), which have been evaluated by calculating harmonic responses, in amplitude and phase, to sinusoidal changes of the ^{14}C production (previous studies[15,25] show a comparison with other models including the Bern2D model). V12b is a hybrid of PANDORA for the ocean[36] and the terrestrial part of the carbon cycle model of Siegenthaler[37]. V12b and V3b were updated to accommodate transient changes in the amount of ^{12}C in all reservoirs (assuming a constant Revelle buffer factor for ocean-atmosphere CO_2 exchanges). The airborne fraction after a thousand years for the 125 PgC release experiments is 0.18 and 0.11 for V3b and V12b, respectively. V3b is thus similar to the BICYCLE model, while version V12b has a carbon uptake faster than BICYCLE or most other models[28]. Sensitivity tests indicate that this problem is partly linked to the short residence time of carbon in the terrestrial biosphere (see below).

For the Suess effect, the time series forcing V12b and V3b (anthropogenic emissions, ^{14}C signature of emissions, natural changes in ^{14}C production) were identical to those forcing BICYCLE (Supplementary Fig. 6a). For the time interval 1820 to 1950 AD, the simulated atmospheric CO_2 rise is 17 and 30 ppmv for

V12b and V3b, respectively (Supplementary Fig. 6b). For the same 1820-1950 period, the atmospheric ^{14}C changes are -21 to $-24‰$ for V12b and V3b, respectively (Supplementary Fig. 6c). The carbon loss from the atmosphere being larger in V12b than in V3b, it is logical to observe that the CO_2 rise and atmospheric ^{14}C depletion are smaller in V12b than in V3b. Increasing the residence time of carbon in the terrestrial biosphere in V12b by a factor five (from 60 to 300 yr) leads to a total CO_2 increase by 24 ppmv and a ^{14}C depletion by $-23‰$ over the $1820 - 1950$ period.

Overall, Supplementary Figs. 6b and 6c show that for the Suess effect V12b and V3b compare well with BICYCLE results and with the CO_2 and $\Delta^{14}C$ observations. The slopes m in the model-data scatter plots (Supplementary Fig. 7a,b) are 0.93 (CO_2 V03b), 0.52 (CO_2 V12b), 0.85 ($\Delta^{14}C$ V03b) and 0.71 ($\Delta^{14}C$ V12b). In particular, V12b and V3b reproduce quite well the natural atmospheric $\Delta^{14}C$ variations before 1900 AD, mainly caused by geomagnetic field and solar activity. Some systematic differences are nonetheless observed: V12b underestimates by 10 ppmv the observed CO_2 rise and by $-5‰$ the ^{14}C depletion in 1950. Surprisingly, V3b matches quite well both observed datasets. As mentioned above, V12b performs better with a longer carbon residence time for its terrestrial biosphere. This effect is reminiscent to what is observed for BICYCLE: CO_2 observations are bracketed by simulations performed with a passive and active terrestrial biosphere, showing a difference of about 12 ppmv in 1950. BICYCLE slightly overestimate atmospheric $\Delta^{14}C$ changes linked to natural forcing.

The anthropogenic CO_2 release until year 2000 was also simulated with these box models, but not the bomb ^{14}C peak. First tests have shown that the post-bomb ^{14}C peak decay in atmospheric $\Delta^{14}C$ in those models is even faster than in BICYCLE, which again is very likely explained by vertical mixing parametrizations between surface and deep ocean. V03b and V12b increased their simulated atmospheric CO_2 between year 1950 and 2000 by about 70 and 40 ppmv, respectively (Supplementary Figs. 6e), leading to slopes m in scatter plot of 1.10 and 0.69 (Supplementary Fig. 7c).

We also performed a model comparison for a generic 125 PgC release over 50 or 200 years with pre-industrial background conditions. The ^{14}C signature of the released carbon was chosen to be $-1000‰$, i.e. ^{14}C-free carbon, equivalent to fossil fuel emissions. BICYCLE (with passive terrestrial biosphere) simulates CO_2 maxima of 37 and 25 ppmv, and $\Delta^{14}C$ minima of -55 and $-23‰$ for the 50 and 200 years release times, respectively (Supplementary Fig. 8). As expected from the Suess effect comparison, V3b produces CO_2 and $\Delta^{14}C$ changes almost as large as BICYCLE while V12b simulates anomalies that are a factor of two smaller. Again, using a longer biospheric residence time in V12b reduces partly the discrepancy (e.g. 23 ppmv and $-38‰$ instead of 18 ppmv and $-34‰$ for the 50 yr release).

For further evaluation these atmospheric carbon cycle perturbations under pre-industrial background conditions were also performed with GENIE, an Earth system model of intermediate complexity, in experiments without climate feedbacks to make them comparable to the box-models. The model configuration of GENIE for this experiment was as described earlier[20] with the addition of a ^{14}C source in the atmosphere that balances the steady-state ^{14}C decay in ocean and atmosphere. In the experiment with 50 years carbon release time the peak amplitudes of $\Delta(pCO_2)$ and $\Delta(\Delta^{14}C)$ in GENIE are $+37$ ppmv and $-54‰$, respectively, so nearly identical to the results in BICYCLE (Supplementary Fig. 8a,c). For the experiment with 200 years release time the amplitudes in GENIE are with $+27$ ppmv ($\Delta(pCO_2)$) and $-26‰$ ($\Delta(\Delta^{14}C)$) slightly larger than in BICYCLE (Supplementary Fig. 8b,d). The decline of the atmospheric carbon anomalies in GENIE are more in agreement with the V03b box model, and slightly slower than in BICYCLE. This agrees with our findings that the airborne fraction f in BICYCLE is on a 100 years time scale comparable

with other models, but on a 1000 years time scale slightly smaller than the multi-model mean[28].

In summary, the comparison of the performances of BICYCLE, and the other models is a useful exercise of model evaluation, giving confidence in the BICYCLE simulations of older carbon cycle perturbations of similar amplitude and timing. We therefore conclude that our carbon cycle model is capable of simulating dynamics in the carbon and ^{14}C cycle of magnitude and duration of the Suess effect. For BICYCLE with passive terrestrial biosphere, the configuration used in the B/A experiments, the relative uncertainties in both CO_2 and Δ^{14}C are 7%, and 10%, respectively. The model thus seems to be a suitable tool for the investigation of the Δ^{14}C anomaly of similar characteristics around 14.6 kyr BP.

Supplementary Note 3

This Supplementary Note describes details of the climate simulations and analysis, which represents the basis for the discussion section that highlights the potential importance of abrupt GHG changes to offset the timing of the temperature maximum leading into the ACR, compared to the onset of the B/A.

(a) Data-based motivation

Ice core data and simulations show that the temperature signals connected with the bipolar seesaw are not uniform across Antarctica. Regional differences of millennial climate variability were found in Antarctic ice cores of different sectors during the last deglaciation[38,39] and MIS 3[40]. These earlier simulation results are not directly applicable for the climate transition around 14.6 kyr BP, because here we have to understand dynamics during an AMOC resumption.

The sequence of bipolar climate changes linking the onset of the B/A and the Antarctic Cold Reversal (ACR) is of particular interest for our study. However, rapid changes in CH_4 are used as age markers to synchronise ice cores from both hemispheres[41] leaving only the independently annual layer-counted chronologies of the WD ice core in the south and of NGRIP in the north for further investigations on the timing of the B/A and the ACR. Note, that temperature change[42] based on a stack from five East Antarctic ice cores (EDC, EPICA DML, Vostok, Dome Fuji, Talos Dome) shows the start of the ACR at approximately the same time as WD (Fig. 5, main text), although the underlying age model is not entirely independent from GICC05. If we analyse water isotopes from the two ice cores WD and NGRIP in detail they reveal a time delayed onset of $\sim$180 years in the ACR[38] relative to the beginning of the B/A[3,43], which is reduced to 145 years, once the Greenland temperature rise was aligned to 14.6 kyr BP. The given maximum counting uncertainties at the onset of the B/A are about 186 years for Greenland cores and 4% or 584 years for the ACR in WD. Note, that these uncertainty estimates already include and capture the suggestion that the GICC05 chronology might be at least 65 years too old around 12 kyr BP[44,45].

(b) Model setup

To test the importance of the abrupt carbon release and associated CO_2 changes for the temperature response in Antarctica we utilise the comprehensive Earth system model COSMOS in a coupled atmosphere-ocean configuration, consisting of ECHAM5[46] and MPI-OM[47] without any flux corrections[48]. The atmosphere

model ECHAM5 was used at T31 resolution ($\sim 3.75°$) with 19 vertical levels. The ocean model MPI-OM was run at an average resolution of $\sim 3°$ with 40 vertical layers. These model components have been applied for a wide range of glacial, interglacial and Neogene applications[8,49,50]. Furthermore, COSMOS has been evaluated with proxy data in an investigation of the LGM[9].

Based on the LGM ocean state and glacial boundary conditions we have performed two experiments in order to investigate the impact of abrupt CO_2 changes on the Antarctic temperature response. In experiment PRE_BA we have simulated 1200 model years to mimic a climate state that is characterised by GHG concentrations prior to the onset of the B/A. The respective concentrations are prescribed by constant values of CO_2 (228 ppmv), N_2O (233 ppbv) and CH_4 (498 ppbv) to represent conditions at ~14.9 kyr BP (reference run). Additionally, we have used simulation PRE_BA as a basis to perform a transient simulation (CO2_ATM) with varying GHG concentrations after 600 years of model integration in PRE_BA.

In the transient experiment CO2_ATM we apply our suggested true atmospheric CO_2 spike of our best guess emission scenario (200 year long release of 0.625 Pg C yr^{-1} (in total 125 PgC) into the atmosphere resulting in a peak amplitude in atmospheric CO_2 change of 22 ppmv) together with measured N_2O [51] and Greenland CH_4 time series, the latter dated according to our understanding (Fig. 5 of main text). All GHG records as taken here are plotted in Supplementary Fig. 5. Using these GHG conditions, CO2_ATM has been run for 600 years. Hence, the two simulations can be used to deduce the impact of GHG changes at the inter-hemispheric temperature signature. Such a GHG forcing scenario seems to be reasonable as suggested by published (Fig. 3a of main text), CO_2 measurements, though absolute values in CO_2 might differ from the ones prescribed in our simulation CO2_ATM.

(c) Climate feedback interpretation

The application of the GHG forcing (Supplementary Fig. 5a) leads to a rise of the annual mean Antarctic surface temperature ΔT_{ANT} and the respective timing of the temperature evolution shows similarities with the temporal signature of the CO_2 forcing. The maximum warming of 0.6 K occurs about 250 years after the onset of the CO_2 rise (Supplementary Fig. 5a). The timing and estimated imprint of this temperature anomaly would generate about +0.5‰ changes in $\delta^{18}O$ if measured in Antarctic ice cores (assuming a slope in the water isotopic thermometer of 0.8‰ per K warming[52]). Based on the inter-hemispheric timing of the ACR in WD (but also in the ΔT_{ANT} stack from East Antarctic ice cores) we can derive that the apparent delay of the maximum isotopic value leading into the ACR with respect to the onset of the B/A in NGRIP can alternatively also be understood as a further increase of temperature and thus of $\delta^{18}O$ in WD (Fig. 5 of main text). The detected increase of ~0.7‰ in WD suggests that the bulk of the observed signal might be explained by the simulated Antarctic temperature response to the abrupt increases in the GHG. The exact contribution will depend on the timing and the strength of the CO_2 pulse.

In this context the amplification of the AMOC at the end of Heinrich stadial 1 and associated temperature changes in Antarctica are of interest here. We thus compare our GHG forcing experiments with results on AMOC strengthening as obtained in previous model simulations using COSMOS[8,9] (Supplementary Fig. 5). Note, that most simulation studies focus on AMOC weakening[40,53]. Furthermore recently, it has been shown that the recovery and amplification of the AMOC in abrupt climate change simulations applying North Atlantic freshwater perturbations is strongly dependent on the climate background state[8]. Therefore, to evaluate the robustness of the Antarctic temperature response (ΔT_{ANT}) we analysed AMOC strenght-

ening for three different climate states that cover a wide spectrum of glacial-interglacial conditions (PI: pre-industrial; LGM: Last Glacial Maximum at 21 kyr BP; MIS3: 32 kyr BP). In these AMOC freshwater hosing experiments, explained in detail elsewhere[8,9], but briefly summarised in the caption to Supplementary Fig. 5, AMOC is weakened for about 150 years by the freshwater perturbation. Subsequently, the end of the freshwater input leads to AMOC resumption within 80–200 years. The magnitude and speed of the AMOC resumption depends on the climate background conditions (Supplementary Fig. 5b), as shown previously[8].

A comparison between the simulated influence of the GHG forcing and the freshwater hosing experiments highlights the relative importance of the GHG induced Antarctic temperature changes (lower panels in Supplementary Figs. 5a,b). As discussed above the GHG forcing might lead to a rise in ΔT_{ANT} of up to 0.6 K after 250 years, depending on the magnitude and timing of the CO_2 pulse. The freshwater hosing experiments are characterised by transient changes in ΔT_{ANT} of less than 0.3 K, starting from the time when freshwater input was shut off. In detail, ΔT_{ANT} depends on the background climate state. For example in the scenario with LGM background conditions ΔT_{ANT} was already rising prior to the freshwater shut-off due to transient changes. Furthermore, a comparison of the transient GHG scenario and the transient AMOC strengthening experiments shows that the ΔT_{ANT} response in the GHG simulation (Supplementary Fig. 5a) is stronger than the simulated impacts by AMOC changes (Supplementary Fig. 5b) already for GHG changes at the time when about half of the the full 22 ppmv increase in CO_2 is reached. Hence, also smaller GHG spikes bear the potential to have a substantial impact on the Antarctic temperature response when compared to effects caused by AMOC changes.

In summary, our model investigations suggest that the abrupt GHG changes are more important for the Antarctic temperature signature than changes associated with an abrupt AMOC strenghening. This highlights the potential of abrupt GHG changes to significantly modulate the Antarctic temperature signature during abrupt climate changes at the end of the last ice age.

Supplementary References

1. Finkel, R. C. & Nishiizumi, K. Beryllium 10 concentrations in the Greenland Ice Sheet Project 2 ice core from 3–40 ka. *Journal of Geophysical Research* **102**, 26699–26706 (1997).

2. Adolphi, F. *et al.* Persistent link between solar activity and Greenland climate during the Last Glacial Maximum. *Nature Geoscience* **7**, 662–666 (2014).

3. Rasmussen, S. O. *et al.* A new Greenland ice core chronology for the last glacial termination. *Journal of Geophysical Research* **111**, D06102 (2006).

4. Reimer, P. J. *et al.* IntCal13 and Marine13 Radiocarbon Age Calibration Curves 0–50,000 Years cal BP. *Radiocarbon* **55**, 1869–1887 (2013).

5. Durand, N. *et al.* Comparison of ^{14}C and U-Th ages in corals from IODP #310 cores offshore Tahiti. *Radiocarbon* **55**, 1947–1974 (2013).

6. Köhler, P., Knorr, G., Buiron, D., Lourantou, A. & Chappellaz, J. Abrupt rise in atmospheric CO_2 at the onset of the Bølling/Allerød: in-situ ice core data versus true atmospheric signals. *Climate of the Past* **7**, 473–486 (2011).

7. Köhler, P., Muscheler, R. & Fischer, H. A model-based interpretation of low frequency changes in the carbon cycle during the last 120 000 years and its implications for the reconstruction of atmospheric Δ^{14}C. *Geochemistry, Geophysics, Geosystems* **7**, Q11N06 (2006).

8. Gong, X., Knorr, G., Lohmann, G. & Zhang, X. Dependence of abrupt Atlantic meridional ocean circulation changes on climate background states. *Geophysical Research Letters* **40**, 3698–3704 (2013).

9. Zhang, X., Lohmann, G., Knorr, G. & Xu, X. Different ocean states and transient characteristic in Last Glacial Maximum simulations and implications for deglaciation. *Climate of the Past* **9**, 2319–2333 (2013).

10. Marland, G., Boden, T. & Andres, R. J. Global, Regional, and National CO_2 Emissions. In *Trends: A Compendium of Data on Global Change* (Carbon Dioxide Information Analysis Center, Oak Ridge National Laboratory, U.S. Department of Energy,, Oak Ridge, Tenn., USA, 2005).

11. Houghton, R. A. Revised estimates of the annual net flux of carbon to the atmosphere from changes in land use and land management 1850-2000. *Tellus* **55B**, 378–390 (2003).

12. Roth, R. & Joos, F. A reconstruction of radiocarbon production and total solar irradiance from the Holocene ^{14}C and CO_2 records: implications of data and model uncertainties. *Climate of the Past* **9**, 1879–1909 (2013).

13. Stuiver, M. & Quay, P. D. Atmospheric ^{14}C changes resulting from fossil fuel CO_2 release and cosmic ray flux variability. *Earth and Planetary Science Letters* **53**, 349–362 (1981).

14. Etheridge, D. M. *et al.* Natural and anthropogenic changes in atmospheric CO_2 over the last 1000 years from air in Antarctic ice and firn. *Journal of Geophysical Research* **D101**, 4115–4128 (1996).

15. Bard, E., Raisbeck, G. M., Yiou, F. & Jouzel, J. Solar modulation of cosmogenic nuclide production over the last millennium: comparison between ^{14}C and ^{10}Be records. *Earth and Planetary Science Letters* **150**, 453–462 (1997).

16. Keeling, R. F., Piper, S., Bollenbacher, A. & Walker, J. Atmospheric CO_2 records from sites in the SIO air sampling network. In *Trends: A Compendium of Data on Global Change.* (Carbon Dioxide Information Analysis Center, Oak Ridge National Laboratory, U.S. Department of Energy, Oak Ridge, Tenn., U.S.A., 2009). doi: `10.3334/CDIAC/atg.035`.

17. Naegler, T. & Levin, I. Closing the global radiocarbon budget 1945-2005. *Journal of Geophysical Research* **111**, D12311 (2006).

18. Hua, Q. & Barbetti, M. Review of tropospheric bomb C-14 data for carbon cycle modeling and age calibration purposes. *Radiocarbon* **46**, 1273–1298 (2004).

19. Grottoli, A. G. & Eakin, C. M. A review of modern coral δ^{18}O and Δ^{14}C proxy records. *Earth-Science Reviews* **81**, 67 – 91 (2007).

20. Cao, L. *et al.* The role of ocean transport in the uptake of anthropogenic CO_2. *Biogeosciences* **6**, 375–390 (2009).

21. Bard, E. Correction of accelerator mass spectrometry ^{14}C ages measured in planktonic foraminifera: Paleoceanographic implications. *Paleoceanography* **3**, 635–645 (1988).

22. Butzin, M., Prange, M. & Lohmann, G. Readjustment of glacial radiocarbon chronologies by self-consistent three-dimensional ocean circulation modeling. *Earth and Planetary Science Letters* **317 - 318**, 177 – 184 (2012).

23. Singarayer, J. S. *et al.* An oceanic origin for the increase of atmospheric radiocarbon during the Younger Dryas. *Geophysical Research Letters* **35**, L14707 (2008).

24. Kubota, K., Yokoyama, Y., Ishikawa, T., Obrochta, S. & Suzuki, A. Larger CO_2 source at the equatorial Pacific during the last deglaciation. *Scientific Reports* **4**, 5261 (2014).

25. Delaygue, G. & Bard, E. An Antarctic view of Beryllium-10 and solar activity for the past millennium. *Climate Dynamics* **36**, 2201–2218 (2011).

26. Woodruff, T., Welten, K., Caffee, M. & Nishiizumi, K. Cosmogenic ^{10}Be in WAIS Divide from 6–19 kyr BP. In *WAIS Divide Ice Core Science Meeting* (24–25 Sep 2013, La Jolla, CA, 2013).

27. Woodruff, T., Welten, K., Caffee, M. & Nishiizumi, K. Cosmogenic ^{36}Cl and ^{10}Be in the WAIS Divide ice core from 6–21 kyr BP. In *AGU Fall Meeting, Abstract C13A-0665* (9–13 Dec 2013, San Francisco, CA, 2013).

28. Joos, F. *et al.* Carbon dioxide and climate impulse response functions for the computation of greenhouse gas metrics: a multi-model analysis. *Atmospheric Chemistry and Physics* **13**, 2793–2825 (2013).

29. Suess, H. E. Radiocarbon Concentration in Modern Wood. *Science* **122**, 415–417 (1955).

30. Köhler, P., Fischer, H., Schmitt, J. & Munhoven, G. On the application and interpretation of Keeling plots in paleo climatic research — Deciphering δ^{13}C of atmospheric CO_2 measured in ice cores. *Biogeosciences* **3**, 539–556 (2006).

31. Köhler, P., Hartmann, J. & Wolf-Gladrow, D. A. Geoengineering potential of artificially enhanced silicate weathering of olivine. *Proceedings of the National Academy of Science* **107**, 20228–20233 (2010).

32. Andres, R. J. *et al.* A synthesis of carbon dioxide emissions from fossil-fuel combustion. *Biogeosciences* **9**, 1845–1871 (2012).

33. Broecker, W. *et al.* How strong is the Harvardton-Bear constraint? *Global Biogeochemical Cycles* **13**, 817–820 (1999).

34. Köhler, P., Fischer, H., Munhoven, G. & Zeebe, R. E. Quantitative interpretation of atmospheric carbon records over the last glacial termination. *Global Biogeochemical Cycles* **19**, GB4020 (2005).

35. Bard, E. *et al.* The North Atlantic atmosphere-sea surface ^{14}C gradient during the Younger Dryas climatic event. *Earth and Planetary Science Letters* **126**, 275 – 287 (1994).

36. Broecker, W. S. & Peng, T.-H. Carbon Cycle 1985: Glacial to interglacial changes in the operation of the global carbon cycle. *Radiocarbon* **28**, 309–327 (1986).

37. Siegenthaler, U., Heimann, M. & Oeschger, H. ^{14}C variations caused by changes in the global carbon cycle. *Radiocarbon* **22**, 177–191 (1980).

38. WAIS Divide Project Members. Onset of deglacial warming in West Antarctica driven by local orbital forcing. *Nature* **500**, 440–444 (2013).

39. Stenni, B. *et al.* Expression of the bipolar see-saw in Antarctic climate records during the last deglaciation. *Nature Geoscience* **4**, 46–49 (2011).

40. Buiron, D. *et al.* Regional imprints of millennial variability during the MIS 3 period around Antarctica. *Quaternary Science Reviews* **48**, 99 – 112 (2012).

41. Veres, D. *et al.* The Antarctic ice core chronology (AICC2012): an optimized multi-parameter and multi-site dating approach for the last 120 thousand years. *Climate of the Past* **9**, 1733–1748 (2013).

42. Parrenin, F. *et al.* Synchronous change in atmospheric CO_2 and Antarctic temperature during the last deglacial warming. *Science* **339**, 1060 – 1063 (2013).

43. Steffensen, J. P. *et al.* High-resolution Greenland ice core data show abrupt climate change happens in few years. *Science* **321**, 680–684 (2008).

44. Muscheler, R. *et al.* Tree rings and ice cores reveal ^{14}C calibration uncertainties during the Younger Dryas. *Nature Geoscience* **1**, 263 – 267 (2008).

45. Lohne, O. S., Mangerud, J. & Birks, H. H. Precise ^{14}C ages of the Vedde and Saksunarvatn ashes and the Younger Dryas boundaries from western Norway and their comparison with the Greenland Ice Core (GICC05) chronology. *Journal of Quaternary Science* **28**, 490–500 (2013).

46. Roeckner, E. *et al.* *The atmospheric general circulation model ECHAM5, part I: Model description*, vol. 349 of *Reports* (Max Planck Institute for Meteorology, Hamburg, Germany, 2003).

47. Marsland, S. J., Haak, H., Jungclaus, J. H., Latif, M. & Röske, F. The Max Planck Institute global ocean/sea ice model with orthogonal curvilinear coordinates. *Ocean Modelling* **5**, 91–127 (2003).

48. Jungclaus, J. *et al.* Ocean circulation and tropical variability in the coupled model ECHAM5/MPI-OM. *Journal of Climate* **19**, 3952–3972 (2006).

49. Knorr, G., Butzin, M., Micheels, A. & Lohmann, G. A warm Miocene climate at low atmospheric CO_2 levels. *Geophysical Research Letters* **38**, L20701 (2011).

50. Zhang, X., Lohmann, G., Knorr, G. & Purcell, C. Abrupt glacial climate shifts controlled by ice sheet changes. *Nature* **512**, 290–294 (2014).

51. Schilt, A. *et al.* Atmospheric nitrous oxide during the last 140,000 years. *Earth and Planetary Science Letters* **300**, 33 – 43 (2010).

52. Masson-Delmotte, V. *et al.* A Review of Antarctic Surface Snow Isotopic Composition: Observations, Atmospheric Circulation, and Isotopic Modeling;. *Journal of Climate* **21**, 3359–3387 (2008).

53. Kageyama, M. *et al.* Climatic impacts of fresh water hosing under Last Glacial Maximum conditions: a multi-model study. *Climate of the Past* **9**, 935–953 (2013).
